# 2D-to-3D Image Reconstruction in Agriculture: A Review of Methods, Challenges, and AI-Driven Opportunities

**DOI:** 10.3390/s26061775

**Published:** 2026-03-11

**Authors:** Hemanth Reddy Sankaramaddi, Won Suk Lee, Kyoungchul Kim, Youngki Hong

**Affiliations:** 1 Department of Computer and Information Science and Engineering, University of Florida, Gainesville, FL 32611, USA; hemanthrsankaram@ufl.edu; 2 Department of Agricultural and Biological Engineering, University of Florida, Gainesville, FL 32611, USA; 3 National Institute of Agricultural Sciences, Rural Development Administration, Jeonju 54874, Republic of Korea; kkcmole@korea.kr (K.K.); sanm70@korea.kr (Y.H.)

**Keywords:** 3D gaussian splatting (3DGS), computer vision, deep learning, LiDAR, neural radiance fields (NeRF), plant phenotyping, precision agriculture

## Abstract

Agriculture is rapidly becoming a data-driven field where automation relies on transforming 2D images into accurate 3D models. However, selecting the most effective method remains challenging due to the unconstrained nature of the environment. This review assesses the effectiveness of geometry-based, sensor-based, and learning-based reconstruction methodologies in agricultural settings. We analyze photogrammetric pipelines, active sensing, and neural rendering methods based on their geometric accuracy, data processing speed, and field performance against wind or occlusion. Our analysis indicates that while Light Detection and Ranging (LiDAR) is highly accurate, it is too expensive for widespread adoption. Conversely, geometry-based methods are inexpensive but struggle with complex biological structures. Learning-based methods, especially 3D Gaussian Splatting (3DGS), have revolutionized the field by enabling a balance between visual fidelity and real-time inference speed. We conclude that the best chance for scalability and accuracy lies in hybrid pipelines that integrate Vision Foundation Models (VFMs) with geometric priors. We believe that “hybrid intelligence” systems, such as edge-native 3D Gaussian Splatting combined with semantic priors, are the future of 3D reconstruction. These systems will enable the creation of real-time, spatiotemporal (4D) digital twins that drive automated decision-making in precision agriculture.

## 1. Introduction

Agriculture is rapidly becoming a data-driven field, with technologies such as automation, artificial intelligence (AI), and computer vision playing a crucial role in managing crops, maximizing yields, and making informed breeding decisions. Most observations today utilize two-dimensional (2D) images, which display color and texture but overlook important geometric information necessary to comprehend biomass distribution, canopy structure, and yield potential. As farming becomes more automated and precise, there is a growing need for systems that can understand plant geometry in three dimensions (3D). This feature enables users to determine the optimal amount of fertilizer to use, monitor crop health, and control pests or weeds in targeted areas [1]. By turning 2D images into accurate digital models, 3D reconstruction helps fill this gap, allowing researchers to better make sense of complex spatial representations [2]. New 2D-to-3D pipelines have been made possible by recent advances in photogrammetry, computer vision, and deep learning. These pipelines are cheaper and more automated than traditional destructive measurements or expensive LiDAR setups. Deep learning advancements, such as transformer-based perception, 3D Gaussian Splatting (3DGS), and Neural Radiance Fields (NeRF), have facilitated the reconstruction of crops, such as soybeans, maize, and wheat, with centimeter-level precision in natural light [3,4,5].

### 1.1. The Imperative in Modern Agriculture

Researchers are struggling to assemble three-dimensional (3D) structures from two-dimensional (2D) images [6]. In agriculture, an accurate comprehension of the morphological and physiological attributes of plants, encompassing plant volume, canopy structure, and leaf area index, forms the basis for data-informed breeding strategies, disease surveillance, and precision crop management [7,8,9]. With the help of 3D reconstructions, robotics, and AI, high-throughput phenotyping systems can extract traits on a large scale and in a continuous manner with minimal human intervention. For instance, automated 3D imaging pipelines have reduced the time required to obtain traits by more than 80% compared to manual methods, while maintaining accuracy within a centimeter [10]. Using Unmanned Aerial Vehicles (UAVs) for multi-view phenotyping, entire crops and fields can be scanned in just a few minutes [11,12]. It generates volumetric and structural datasets closely linked to indices of biomass, yield, and stress tolerance [13,14]. Deep learning models use 3D data to combine canopy architectural phenotypes in wheat [15]. Additionally, precision agriculture utilizes 3D canopy models to optimize variable-rate spraying, which minimizes environmental impact by applying chemicals based on foliage volume rather than land area. These changes demonstrate that high-throughput, 3D-enabled phenotyping is not only a technological breakthrough but also a biological necessity for achieving the goals of precision and sustainable agriculture, expanding breeding programs, and precise farming [15].

### 1.2. Limitations of Traditional and 2D Methodologies

**Figure 1:**
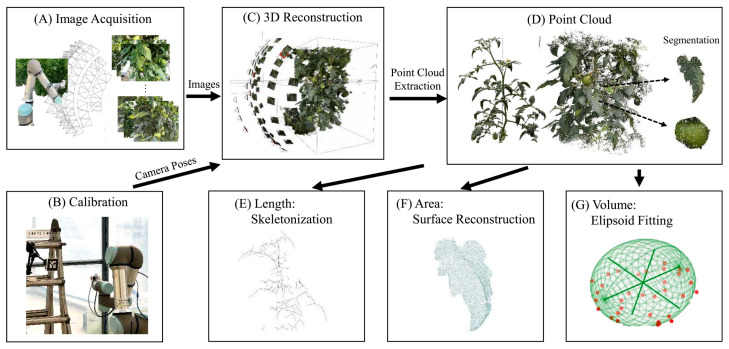
Agriculture-oriented multi-view 3D reconstruction workflow. The pipeline progresses from (**A**) image acquisition and (**B**) calibration/camera pose estimation to (**C**) 3D reconstruction, followed by (**D**) point-cloud extraction and segmentation. The reconstructed geometry is then used for quantitative phenotyping, including (**E**) skeleton-based length estimation, (**F**) surface reconstruction for area measurement, and (**G**) volume estimation via ellipsoid fitting [11].

Traditional methods, such as manual measurement and destructive sampling, can provide helpful information; however, they are time-consuming and labor-intensive, making them impractical for large-scale projects [16]. Because a 2D image is a flat projection of a complex 3D world, it can be challenging to determine the size of an object and its distance from the camera. This makes it hard to get the right size and volume. Two-dimensional imaging has enabled the non-invasive monitoring of plant traits; however, it has several limitations, including dense canopies, occlusion, and changing lighting, which make it challenging to capture complex structures and accurately estimate volume [17,18]. In field conditions, 2D imaging is affected by occlusion, changing light, and plant organs that overlap, which makes the measurements less reliable. As crop canopies become thicker later in their growth, the problems we discussed earlier worsen, making it harder for 2D imaging systems to accurately record volumetric biomass and complex structures. Due to this, 2D methods are still valid for initial screening; however, they lack the geometric accuracy and scalability required for modern high-throughput phenotyping and precision crop production. Consequently, even sophisticated 2D indices, such as NDVI and Excess Green, fail to accurately capture true volumetric biomass or structural complexity, a limitation that is particularly pronounced in multi-layered canopies. The fundamental process of recovering 3D structure from 2D imagery via multi-view geometry, camera calibration, and point-cloud reconstruction, and subsequently extracting plant traits (e.g., length, surface area, and volume) is illustrated in the workflow shown in Figure 1.

Different growth patterns and spatial distributions make analysis even more complicated. Researchers and farmers both require detailed plant metadata to effectively manage crops, achieve optimal yields, and make informed breeding decisions. Thus, it is essential to address the shortcomings of conventional 2D methodologies and to identify the most efficient 3D reconstruction techniques for agricultural applications, thereby improving existing practices and investigating innovative methods for future progress. Furthermore, intricate plant-environment interactions essential for modeling photosynthesis and resource utilization, such as leaf angle dynamics, canopy porosity, and radiation interception efficiency, cannot be accurately quantified by 2D imaging. The absence of spatial depth limits the integration of multimodal data that collectively characterize plant productivity and health, including spectral, thermal, and structural indicators. As agricultural systems transition toward automation, robotics, and digital twins, three-dimensional data is becoming increasingly crucial for high-fidelity growth modeling and decision support [19]. In particular, accurate 3D canopy maps are needed for precision spraying and variable-rate application systems to find target areas for nutrients, herbicides, or pesticides. This ensures that resources are used efficiently and that the environment is protected. 3D reconstruction enables the complete retrieval of morphological, textural, and volumetric data from conventional 2D imagery, thereby representing a logical advancement in image-based phenotyping in this context.

### 1.3. The Emergence of 3D Reconstruction as a Solution

These problems have made 3D reconstruction more popular because it can provide accurate and complete representations of how plants appear. Various strategies have been investigated for 3D model reconstructions derived from 2D inputs [20]. LiDAR, Computed Tomography (CT), and Magnetic Resonance Imaging (MRI) are examples of sensor-based systems that can create very accurate reconstructions. However, they are costly and do not work well in the field [21,22]. Geometry-based methods, such as multi-view stereo and structure-from-motion pipelines, have yielded promising results in crops such as maize and lettuce [23,24]. Nonetheless, difficulties arise in addressing canopy complexity and occlusion. Computer vision and artificial intelligence have created new paths. Learning-based methods, such as neural implicit surfaces [25], Plant-NeRF for natural-light reconstructions, and transformer-based models such as the Visual Geometry Grounded Transformer (VGGT) [26], demonstrated the capability of AI to capture structural details in field conditions accurately. Zero-shot frameworks, such as Segment Any Leaf 3D [27], have made 3D reconstruction more useful by enabling leaf segmentation and reconstruction without requiring extensive data. AI pipelines can sometimes achieve the same level of accuracy as sensor-based systems that utilize expensive LiDAR or CT scanners, but they only require standard images in controlled settings. For instance, Plant-NeRF achieved reconstruction errors of less than 0.8 cm RMSE, whereas VGGT’s point-cloud accuracy was within 2–3% of the LiDAR benchmarks. In addition to their accuracy, these pipelines are inexpensive, scalable, and easier to deploy in agricultural settings. Three-dimensional trait measurements enhance fungicide scheduling, facilitate breeders’ selection of disease-resistant cultivars, and enable researchers to monitor plant growth under stress [28]. The aim of this review is to provide a comprehensive overview of the various methods for transitioning from 2D to 3D, examine their advantages and disadvantages, and explore how AI-powered methods can enhance the measurement of agricultural traits. There are talks about pipelines based on geometry, sensors, learning, and a mix of the three. There are also discussions about common processing workflows that span from acquiring images to generating point clouds and estimating volume. There are also trade-offs between cost, accuracy, and scalability. The analysis concludes by emphasizing opportunities such as multimodal data fusion, lightweight learning models, and real-time, field-ready systems, while also pinpointing significant challenges that hinder field implementation.

### 1.4. Search Strategy and Selection Criteria

The literature search was conducted following a PRISMA-style approach to identify studies on 2D to 3D reconstruction. Publications from 2020 to 2025 were retrieved from Google Scholar, Web of Science, Scopus, IEEE Xplore, ScienceDirect, and Frontiers.

The following search strings were used by combining agriculture and reconstruction keywords:

(“plant phenotyping” OR “precision agriculture” OR “crop monitoring”) AND (“3D reconstruction” OR “SfM” OR “MVS” OR “SLAM” OR “LiDAR” OR “RGB-D” OR “NeRF” OR “3D Gaussian Splatting” OR “implicit surface” OR “DUSt3R” OR “VGGT”). Studies were included if they reconstruct 3D geometry from 2D imagery, with or without auxiliary depth information, target applications such as crop, plant, or field-level reconstruction and monitoring, present computer vision methodologies transferable to agricultural contexts, and provide quantitative evaluation through reported results or reproducible pipelines. Studies were excluded if they focused on non-agricultural domains without methodological relevance, were non-English, non-peer-reviewed, or lacked accessible full-text availability. After screening approximately 210 papers, 173 studies covering geometry-based, sensor-based, learning-based, and hybrid approaches were retained. Following key reviews, additional sources were identified.

## 2. Approaches to 2D-to-3D Reconstruction

The approaches are grouped into geometry-based, sensor-based, learning-based, and hybrid/fusion methods. Each pathway offers unique advantages and limitations in terms of cost, accuracy, and scalability.

### 2.1. Geometry-Based Methods

Geometry-based pipelines rely on principles of multi-view geometry to reconstruct 3D structure from overlapping images. Common techniques include:

#### 2.1.1. Structure-from-Motion (SfM)

Structure-from-Motion (SfM) is a photogrammetric method that simultaneously estimates both the 3D shape of a scene and the camera positions [29]. The first step is to get unique feature points from a series of overlapping images, heavily relying on scale-invariant depth estimation [30]. These points can be SIFT, SURF, or ORB descriptors. Next, these features are matched across different views to create 2D–2D correspondences. These correspondences are then used to figure out how the cameras are moving relative to each other using epipolar geometry. Using the fundamental or essential matrix, SfM finds the relative translation and rotation of each image pair. These values are then refined using global optimization through bundle adjustment (BA), an iterative process that minimizes the reprojection error between the observed and predicted image points [31]. Triangulating matched features creates a sparse point cloud that shows the object’s or scene’s structural framework. SfM has been demonstrated to be an effective and cost-effective method for rebuilding large field plots using UAV or ground-based imaging in agriculture [32,33,34]. The digital surface and canopy height models developed through this work have been utilized to study crop growth, estimate biomass, and predict yields. However, SfM accuracy can be affected by motion blur, repeating leaf textures, and wind-caused movement of the canopy, which can cause features to misalign or cause changes in shape. Even with these problems, the low cost of the equipment and its versatility make SfM one of the most important methods for 3D plant phenotyping.

#### 2.1.2. Multi-View Stereo (MVS)

**Figure 2:**
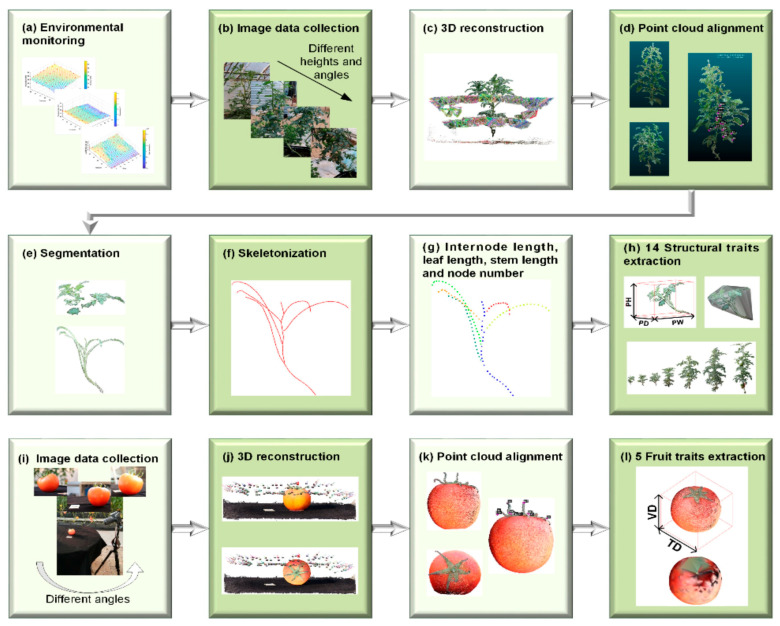
Agriculture-focused 3D phenotyping workflow based on multi-view reconstruction. The pipeline progresses from (**a**) environmental monitoring and (**b**) multi-angle image collection to (**c**) 3D reconstruction and (**d**) point-cloud alignment, followed by (**e**) segmentation and (**f**) skeletonization for structural analysis. Plant architectural traits (e.g., internode/leaf/stem length and node count) and additional structural traits are then extracted (**g**,**h**). A parallel branch demonstrates fruit phenotyping from multi-angle imaging through reconstruction and alignment to fruit-trait extraction (**i**–**l**) [25].

Multi-View Stereo (MVS) works with SfM to turn sparse reconstructions into dense, detailed 3D models [35,36]. After SfM has determined the camera poses and a rough point cloud, MVS algorithms create dense depth maps by identifying pixel-level matches between multiple overlapping images. These correspondences are established along epipolar lines to ensure consistent geometry. The differences are then triangulated to get depth values for each pixel. Then, the individual depth maps from different angles are combined into one dense point cloud or triangular mesh. This is typically achieved using volumetric or Poisson surface reconstruction methods to maintain a smooth surface. To make the model look more real and easier to understand, textures are projected back onto it. Multi-view stereo (MVS) based dense reconstruction is used as part of the multi-view pipeline to generate detailed 3D point clouds for subsequent alignment, segmentation, and trait extraction, as summarized in Figure 2 [37].

MVS is especially useful for rebuilding fragile canopy structures, single leaves, and plant organs in controlled or field conditions in farming settings, such as for mature soyabeans [38]. For example, dense 3D reconstructions have been used to measure the area of leaves, the porosity of the canopy, and the height difference with millimeter-level accuracy [39,40]. MVS has trouble in the field due to factors such as overlapping leaves, changing sunlight, and reflective surfaces, which can make pixel matching less accurate. Still, MVS is one of the easiest high-accuracy reconstruction methods to use when combined with poses from SfM [41].

#### 2.1.3. Convex and Concave Hull Approximations

Convex and concave hull algorithms offer simplified geometric approximations for estimating plant volume and spatial occupancy. The convex hull is the smallest convex polyhedron that contains all the 3D points. The concave hull, on the other hand, adds some inward curvature to better match the object’s true outline. You can figure out the volume inside by doing math or using numerical integration. This provides a quick way to determine the amount of biomass or canopy density present. These methods are efficient for computers, so they can be used for real-time monitoring or high-throughput phenotyping systems where rough volumetric measurements are enough. However, they may not accurately determine the true plant volume, especially for species with hollow or complex internal structures. Even though hull-based methods are simple, they are useful for pre-processing or benchmarking more advanced volumetric analyses.

#### 2.1.4. Voxel Carving and Silhouette-Based Models

Voxel carving and silhouette-based reconstruction are traditional computer vision techniques that infer 3D shape from segmented 2D silhouettes obtained from various perspectives [42]. The first step is to separate the object (the plant) from the background in each picture using thresholding, chroma-keying, or semantic segmentation. Then, a 3D voxel grid is set up to surround the object’s approximate space. The image planes display each voxel, and any voxel that does not fit within the silhouette region in any view is removed (“carved”). After considering all the views, the remaining voxels come together to form the visual hull, which is a rough estimate of the object’s outer surface.

Voxel carving is a non-destructive method for determining the volume of a plant’s canopy, the distribution of leaf angles, and the shape of the plant’s symmetry in a greenhouse. It is appealing for controlled-environment studies because it is computationally efficient and requires few sensors. However, it cannot capture concave areas or hidden internal structures, which makes it less useful for complicated field scenes. Even so, silhouette-based methods are helpful for teaching and testing in 3D plant modeling because they are easy to understand and use.

#### 2.1.5. Stereo Imaging with SfM and Point Cloud Registration

Stereo imaging, SfM, and point cloud registration work together to make a hybrid geometry-based pipeline that improves the accuracy of reconstruction for complicated plant structures [43]. In this workflow, pairs of high-resolution stereo images are taken from fixed angles [44]. Then, traditional stereo correspondence algorithms are used to make local disparity maps [45]. Then, SfM improves these local point clouds to find the global camera poses and how the views are lined up with each other [46]. To make sure that the geometry is consistent, individual point clouds are combined using the Iterative Closest Point (ICP) registration [47]. This starts with rough alignment based on known calibration targets, such as spheres or checkerboards, and then gets better with each iteration based on point-to-point distances. The combined 3D model is meshed and smoothed to take a continuous, high-quality picture of the plant [48]. The “binocular + SfM + ICP” pipeline has been used in agriculture to rebuild crops with complex branching structures, such as maize, rapeseed, and grapevine [49,50]. It has an accuracy of less than a centimeter and reduces the distortions that are common in systems that only use stereo or monocular vision [51]. But the method needs careful calibration and stable lighting while capturing, and it can take a long time to process large datasets [52,53]. Even with these problems, this hybrid geometric approach strikes a great balance between cost, accuracy, and detail, making it perfect for phenotyping applications that need very precise structures.

### 2.2. Sensor-Based Methods as Benchmarks and Fusion Sources

Sensor-based techniques are essential because they supply the geometric “ground truth” needed to validate photogrammetric and neural pipelines, even though the focus of this review is inferring 3D structure from 2D imagery [54]. Additionally, active sensors are being used more frequently in hybrid workflows to address occlusion problems and scale ambiguities that are present in pure 2D-to-3D approaches [55].

#### 2.2.1. LiDAR (Light Detection and Ranging)

**Figure 3:**
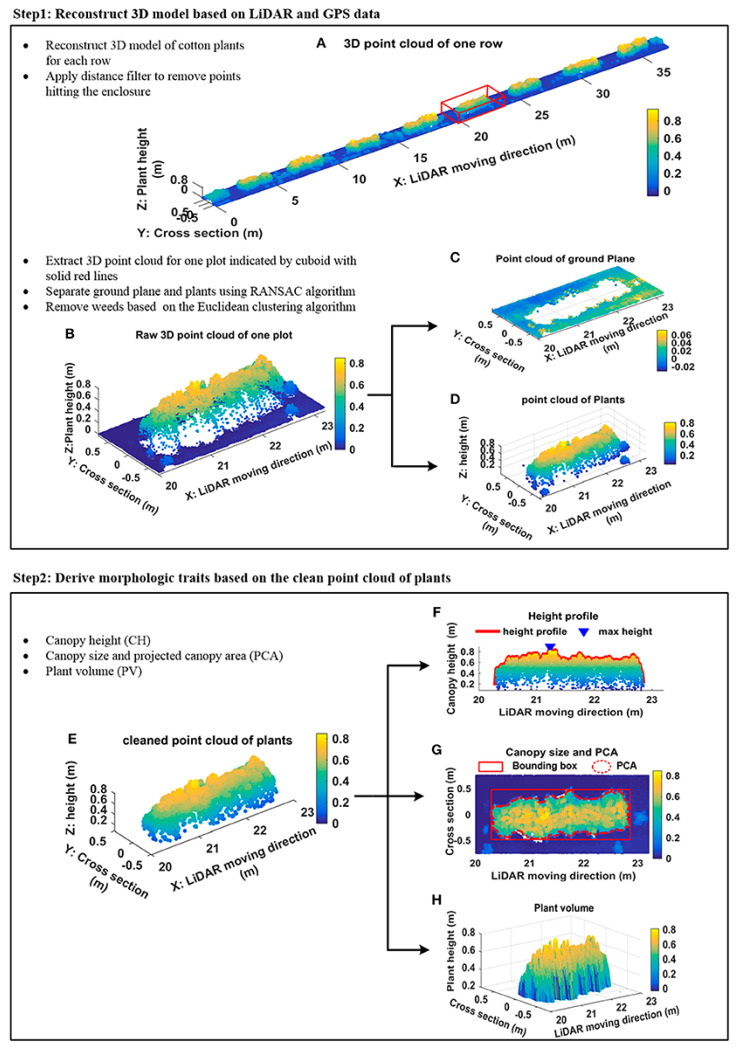
LiDAR pipeline for plot-level canopy trait estimation. (**A**) 3D point cloud of a scanned row. (**B**) Plot extraction via cuboid ROI. (**C**) Ground-plane point set. (**D**) Plant point set. (**E**) Denoised plant point cloud. (**F**) Height profile and maximum canopy height. (**G**) Projected canopy area. (**H**) Plant volume [29].

LiDAR works by sending out short laser pulses toward a target and measuring the time it takes for the light to return. This time is used to figure out the distance [56]. Every pulse has exact angular and positional metadata, which creates a dense “point cloud” where each point has 3-D Cartesian coordinates (x, y, z) and often information about how bright the surface is [57]. Modern LiDAR systems use scanners that rotate or oscillate to scan the scene. These scanners can send data at rates of hundreds of thousands of points per second [58]. LiDAR mounted on terrestrial tripods, ground vehicles, or UAVs can measure the height of crops, the shape of their canopies, and other structural traits, even when the foliage is only partially covering them [59,60]. LiDAR is great for estimating biomass and volume because near-infrared laser light can go through leaf layers and accurately measure structures both above and below the canopy [61]. LiDAR units, on the other hand, are expensive and can be affected by motion caused by wind, so they need to be carefully calibrated and registered between scans [62,63]. Figure 3 shows an example of canopy trait estimation using LiDAR.

#### 2.2.2. Time-of-Flight (ToF) and RGB-D Cameras

**Figure 4:**
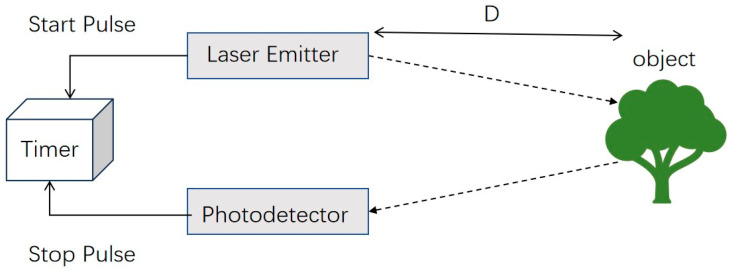
Schematic of a Time-of-Flight (ToF) system, highlighting the pulse-return principle where a laser emitter and photodetector measure the distance (D) to an object, such as a tree, for rapid depth capture [31].

Time-of-Flight and RGB-D sensors are small, cheap options for capturing 3D images at close range [64]. To get depth, they project modulated infrared light onto a scene and measure either the phase shift or the round-trip time delay of the reflected signal at each pixel (Figure 4) [65]. The outcome is a depth map synchronized with a color (RGB) image, facilitating integrated geometric and visual analysis [66]. Commercial cameras such as Intel RealSense (Intel Corporation, Santa Clara, CA, USA) or Microsoft Azure Kinect (Microsoft Corporation, Redmond, WA, USA) do this in real time, making depth data at 30–90 frames per second with millimeter-level accuracy at distances of up to ~3 m [67]. The internal workflow includes calibrating the emitter, fixing the depth for multi-path interference, and combining RGB images with intrinsic/extrinsic calibration matrices [68]. For agricultural purposes, these sensors are good for greenhouse or lab phenotyping, where light can be controlled, and the distance to the target is limited [69]. They are portable and fast, but they do not work well in bright sunlight, on reflective leaf surfaces, or in long-range field conditions where the infrared projection strength is too weak [70,71]. Figure 4 illustrates the schematic of a ToF system measuring the distance to a tree, highlighting the pulse-return principle used for rapid depth capture.

#### 2.2.3. Tomographic Imaging (CT, MRI, X-Ray)

Computed Tomography (CT), Magnetic Resonance Imaging (MRI), and X-ray micro-CT are all types of tomographic modalities that make 3D models of internal structures by taking many cross-sectional images and turning them into a volumetric model [72]. In CT, a rotating X-ray source takes attenuation profiles through the object. The Radon transform of these projections is mathematically inverted, usually using filtered back-projection or iterative reconstruction to make a 3-D density map [73]. MRI, on the other hand, measures proton spin resonance in a magnetic field. This gives it a soft tissue contrast without using ionizing radiation [74]. You can export the volumetric grids in standard DICOM format so that you can work on them more [75]. In plant science, these techniques facilitate non-destructive imaging of root systems, stems, and internal fruit tissues, elucidating moisture distribution and structural integrity [76]. CT and MRI machines have very high spatial resolution (tens of micrometers), but they are large, expensive, and need controlled environments. This means that they can only be used for laboratory phenotyping and not for open-field studies [77].

#### 2.2.4. CT-Based Reconstruction Workflow

**Figure 5:**
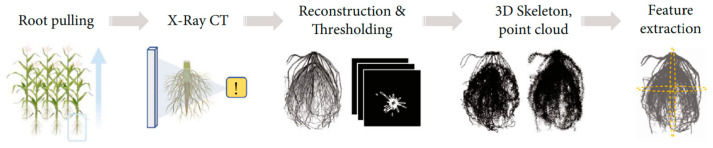
Pipeline for 3D root imaging using X-ray Computed Tomography, detailing the progression from root pulling and radiographs to 3D skeleton point cloud generation and volumetric feature extraction [33].

A complete tomographic reconstruction has several important steps [78]. First, contrast-enhanced scanning gets raw projection data, which makes hundreds or thousands of thin 2-D slices. These slices, which are usually in DICOM format, are prepared ahead of time to get rid of noise and fix artifacts such as beam hardening and ring effects [79]. After that, the data is brought into specialized reconstruction software, usually 3D Slicer or commercial medical platforms. There, threshold-based or machine-learning segmentation separates areas of interest, such as vascular bundles, roots, or internal voids [80]. To make a volumetric model, segmented slices are stacked and interpolated. Then, marching-cubes algorithms are used to mesh the surface [81]. You can measure the internal porosity, tissue density, or structural changes in the 3-D object that you made [82]. CT-based workflows are very demanding on computers, but they are the best way to measure root phenotypes and find internal defects in crops [83,84]. The progression from raw radiographs to 3D skeleton point cloud generation and volumetric feature extraction is detailed in the X-ray Computed Tomography pipeline in Figure 5.

#### 2.2.5. Multi/Hyperspectral + Depth Sensors

**Figure 6:**
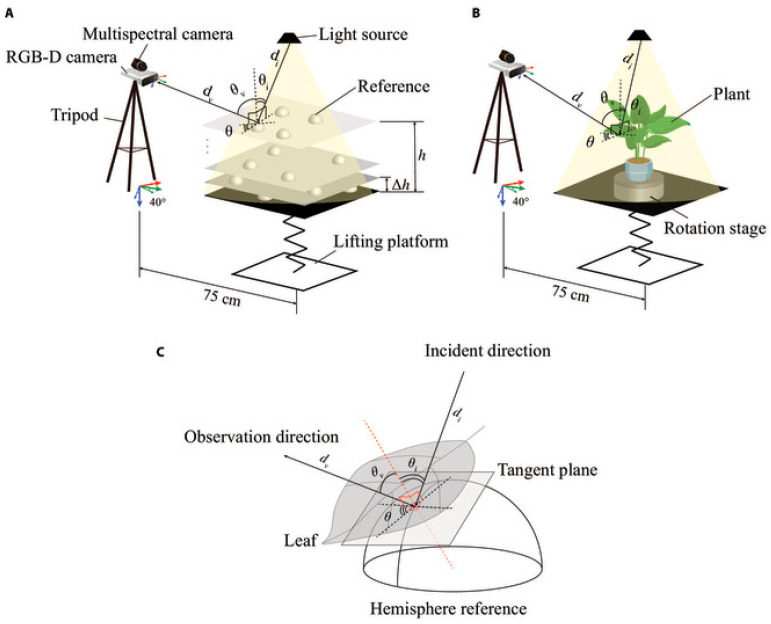
Experimental setup for a multi-sensor imaging system, illustrating the synchronized acquisition of RGB-D and multispectral data to determine 3D light field features of plant leaves. The system components include: (**A**) The reference measurement setup, utilizing a lifting platform with reference targets to calibrate height and distance; (**B**) The plant imaging configuration, employing a rotation stage to capture multi-angle views of the specimen; and (**C**) A geometric representation detailing the incident light direction, observation direction, and corresponding angles relative to the leaf’s tangent plane [35].

Hyperspectral imaging goes beyond regular RGB imaging by capturing reflectance across tens or hundreds of narrow spectral bands, usually between 400 and 1000 nm [85]. When used with depth sensors, it makes a spectro-geometric dataset that connects physical structure with biochemical composition [86]. The system gathers synchronized hyperspectral cubes and depth maps. Radiometric calibration changes raw digital numbers into surface reflectance, and geometric calibration matches each spectral pixel with its 3-D coordinate [87]. This combination of different types of data lets us estimate chlorophyll, water, and nitrogen levels in real 3-D space, which makes it possible to assess stress or nutrients in a specific area [88]. Hyperspectral-depth fusion has been used in real life to find early signs of disease or keep an eye on canopy heterogeneity in the field [89]. The biggest problems are getting the co-registration right and dealing with a lot of data, which needs careful wavelength-dependent calibration and computing power for real-time use [90,91]. Figure 6 demonstrates the experimental setup for a multi-sensor imaging system, illustrating the synchronized acquisition of RGB-D and multispectral data.

#### 2.2.6. Azure Kinect RGB-D Scanning

Teng et al. (2021) [36] showed a controlled-environment phenotyping workflow that used the Microsoft Azure Kinect RGB-D camera to scan rapeseed plants throughout their entire growth cycle. The system takes synchronized RGB, depth, and infrared pictures from six different angles around each plant [92]. Spatial and temporal filtering are used to clean up depth maps before they are used. A multi-stage Iterative Closest Point (ICP) algorithm is used to down sample and register each partial point cloud [93]. The first step is coarse alignment based on rough camera geometry, and the second step is fine alignment that optimizes the Euclidean distances between overlapping points [94]. Voxel-grid averaging is used to make the density and smoothness even, which results in a coherent, colorized 3-D model [95]. The datasets that come from this make it possible to accurately measure plant height, leaf angle, and branching angle over time [96]. This workflow shows how medium-sized crops can get near-LiDAR accuracy from off-the-shelf RGB-D cameras when they are used with strong calibration and registration methods [97].

#### 2.2.7. Single-Pixel Imaging (SPI)

**Figure 7:**
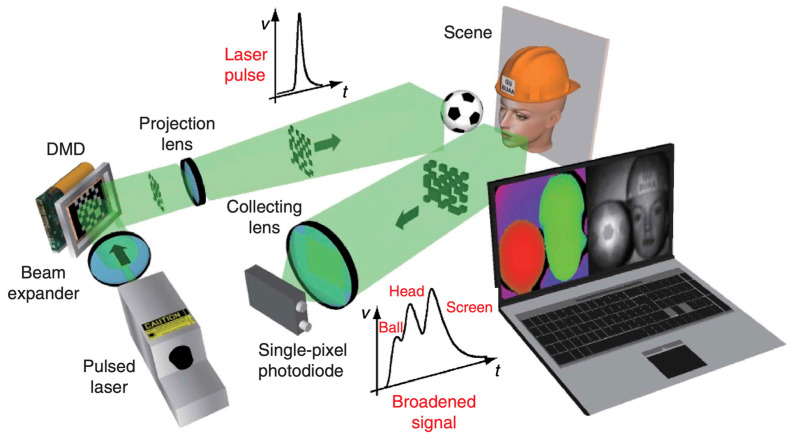
Schematic of a Single-Pixel 3D Imaging system, utilizing a pulsed laser and Digital Micromirror Device (DMD) to reconstruct depth and reflectivity from back-scattered light intensity [38].

Single-Pixel Imaging is a computational imaging approach that reconstructs 2-D or 3-D scenes utilizing a single photodetector instead of a pixelated sensor [98]. The single detector records the integrated reflected intensity for each structured light pattern, which is usually a Fourier or Hadamard basis function [99]. Then, by using compressive sensing or inverse Fourier transform methods, these measurements are compared to the known lighting patterns to make a 3-D reconstruction [100]. The new 3-D Underwater Fourier Single-Pixel Imaging (3D-UFSPI) system showed that Fourier basis projection keeps spatial fidelity even when there is a lot of scattering [101]. It was able to make accurate 3-D surfaces at sampling rates as low as 15% [102]. In farming, SPI’s ability to work through dirty or cloudy areas and its resistance to optical noise make it a good choice for specialized uses such as looking at the soil-root interface or checking fruit below the surface [103]. Even though the acquisition speed is slower than that of array cameras, SPI shows how computational optics can get around the problems that traditional sensors have in tough situations [104,105]. The methodology for utilizing a pulsed laser and Digital Micromirror Device to reconstruct depth and reflectivity is shown in the Single-Pixel 3D Imaging schematic in Figure 7.

#### 2.2.8. Structured Light Scanning

**Figure 8:**
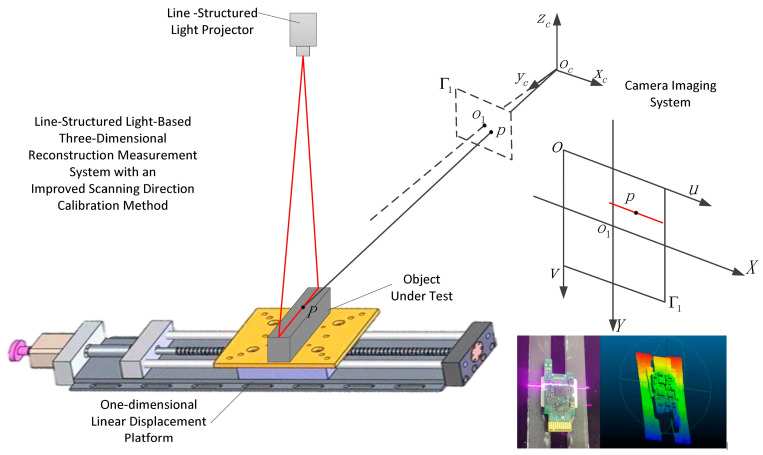
Principle of line-structured light scanning for 3D reconstruction, where a calibrated projector casts a light stripe to recover surface geometry via triangulation in controlled environments [39].

Structured Light scanning is another way to actively sense something [106]. It shines a known pattern of light, such as lines, grids, or stripes, onto an object [107]. This pattern changes shape when it hits the object’s surface, and a camera captures this change [108]. The system can figure out the 3D coordinates for each point on the surface by using triangulation to look at this deformation [109]. This creates a high-resolution point cloud. This method is well known for being very accurate and able to pick up small details on surfaces [110]. This makes it good for close-range scanning of single plant organs or seedlings in controlled settings [111]. Structured light scanners, on the other hand, are very sensitive to light in the environment, especially sunlight [112]. This can make the projected pattern too bright and make it hard to use in the field [113]. They are also usually only good for short-range uses [114]. The principle of line-structured light scanning for recovering surface geometry via triangulation in controlled environments is presented in Figure 8.

### 2.3. Hybrid/Fusion Pipelines

Hybrid methods combine multiple data modalities to balance robustness and cost.

#### 2.3.1. Sensor + Geometry Fusion

A practical field pipeline first synchronizes LiDAR and RGB, calibrates extrinsics (target or target-free), and uses SfM to get the camera poses and a rough, scale-ambiguous cloud [115]. Then, sparse LiDAR ranges are projected into the images to (i) fix absolute scale in bundle adjustment, (ii) seed/regularize MVS depth with reliable anchors in low-texture foliage, and (iii) fill self-occluded canopy regions that photogrammetry misses [116]. The fused depths are combined with volumetric methods (such as TSDF) to make watertight, textured meshes that can be used to extract traits [117]. LiDAR intensity can also help separate leaves from wood [118]. In orchards and row crops, this co-fusion usually cuts down on drift, makes the area under the canopy more complete, and keeps reconstructions stable even when the lighting changes and the textures are repeated, which is where SfM/MVS alone has trouble [119]. LiDAR-inertial SLAM gives strong poses on mobile rigs that make RGB alignment even tighter before fusion [120]. This makes long plots more accurate and easier to scale [121]. Wind-induced misregistration, mixed-pixel effects on thin leaves, and calibration drift are some of the most common problems that can happen during fusion [122]. Frequent extrinsic checks, motion compensation, and depth-confidence weighting can help reduce these problems [123].

#### 2.3.2. Learning + Geometry

In practice, hybrid pipelines “clean” inputs for classical geometry with learned front ends, such as semantic/sky–soil–leaf segmentation to suppress outliers, deblurring/denoising to stabilize features, and monocular depth priors to warm-start dense matching [124]. This way, SfM/MVS sees sharper key points and fewer leaf-motion artifacts [125]. The opposite direction is just as strong: geometric tools give neural models a stable and fast base SfM (or DUSt3R/MASt3R) gives camera poses and rough geometry so that radiance- or Gaussian-based learners can train with little optimization for each scene [126]. Ag-specific systems are a good example of this synergy: OB-NeRF uses SfM poses for complex plants to cut down on pose drift and speed up convergence [127]. Plant-NeRF uses multi-view geometry to hold reconstructions steady in natural light [128]. Recent Gaussian pipelines go even further by combining SAM-style masks or foundation features with pose/patch geometry to get clear boundaries and make training at field scale easier [129]. Downstream, geometry-aware fusion (such as TSDF/mesh extraction) turns the learned fields into watertight, trait-ready meshes. It does this by using learned confidence to weight unreliable leaves or sun-glare areas [130]. The net effect is that there are fewer gross failures caused by light and wind, training goes faster, and there is more completeness in under-canopy areas than with either learning or geometry alone [131].

#### 2.3.3. Multimodal Fusion

This pipeline combines complementary sensors, RGB for texture, hyperspectral for chemistry, thermal for energy balance, and depth/LiDAR for metric structure into a single 3D frame so that each vertex/voxel has both geometry and physiology [132]. A standard workflow synchronizes streams in time, calibrates the radiometric (per-band reflectance/thermal emissivity) and geometric (intrinsics/extrinsics) properties, projects spectral/thermal pixels onto SfM/MVS or LiDAR geometry, and fuses them volumetrically (e.g., TSDF/mesh) or through learned cross-modal features [133]. In practice, LiDAR + RGB/MVS improves completeness and metric accuracy when there is wind or something is blocking the view [134]. Hyperspectral + depth, on the other hand, gives you chlorophyll, water, and nitrogen maps directly “in 3D,” which lets you ask questions about traits such as canopy porosity with stress indices that are in the same place [135]. Recent plant pipelines combine vision foundation models or SAM-style masks with 3D Gaussian Splatting to make modalities fit together and make organ boundaries clearer across scenes [136]. Cross-sensor parallax, wavelength-dependent distortion, and scale drift are major problems; solutions include per-band distortion models, learned co-registration, and LiDAR-anchored scaling [137]. The end result is better, trait-ready reconstructions that connect structure to function for strong field phenotyping [138].

#### 2.3.4. Active View Planning (SSL-Local-NBV)

Self-Supervised Learning Local Next-Best-View (SSL-Local-NBV) is a self-supervised next-best-view planner that learns to score candidate viewpoints based on their expected information gain, which is usually measured by how much the depth/occupancy uncertainty goes down or how much the newly visible surface goes up when the incremental reconstruction (such as SfM/MVS or 3D Gaussian Splatting) is updated [139]. A lightweight view-trajectory network regularizes selections to avoid redundant overlaps and enforce smooth, short motion, while a receding-horizon policy picks poses that jointly maximize gain and minimize travel cost under field constraints (wind, occlusion, narrow rows) [140]. The policy adapts across species and growth stages because supervision comes from on-the-fly reconstructions and visibility simulations instead of labels. It can also be added to existing plant-mapping stacks to cut images while improving coverage and completeness [141]. Complementary research on agricultural view planning (BOSfM) demonstrates analogous advantages from information-gain criteria and motion-aware pathing, emphasizing that learned NBV surpasses fixed rings or lawn-mower scans in congested canopies [142]. Recent evaluations of 3D pipelines show that NBV is a key factor for robustness and cost, especially when combined with LiDAR/MVS or Gaussian splats. This is because it targets occluded organs instead of oversampling easy views [143]. In practice, results indicate enhanced surface coverage with reduced views and abbreviated scan durations in both simulation and greenhouse/field trials, establishing SSL-Local-NBV as an effective active-sensing module for high-throughput phenotyping [144].

#### 2.3.5. Truncated Signed Distance Fusion (TSDF)

TSDF combines multiple depth maps either from active sensors or trained neural models into a voxel grid that stores the truncated signed distance to the nearest surface. It then uses confidence weights and camera poses to remove noise, fill in small holes, and create a smooth implicit surface that is later meshed (usually using Marching Cubes) and textured from the aligned RGB [145]. In practice, three knobs control the trade-off between quality and cost: voxel size (which sets the geometric resolution), truncation band (which controls smoothing vs. detail), and per-pixel weighting/pose accuracy (which causes ghosting when there is drift or mis-calibration). This makes TSDF a strong last step for turning multi-view predictions into watertight assets for trait computation [146]. When depth comes from learned radiance or explicit field renderers such as NeRF or 3D Gaussian Splatting, TSDF makes it easy to separate view synthesis from surface extraction. You can render depths from new views, combine them volumetrically, and then get a consistent mesh for measurement [147]. Agricultural pipelines say that this method makes completeness and texture consistency better when there are few or uneven viewpoints [148]. They also say that memory limits on large fields (which can be fixed by using chunked or gridded volumes) and the risk of over smoothing very thin organs at coarse voxel sizes [149].

#### 2.3.6. Generative Adversarial Networks (GANs) for Sub-Tasks

In hybrid 2D to 3D pipelines, GANs are best used as helpers that clean and enrich inputs before geometry is solved, e.g., semantic segmentation of structures/leaves (masking clutter for SfM/MVS), illumination normalization and dehazing for outdoor crops, super-resolution/denoising of RGB or depth maps, and inpainting thin organs to stabilize meshing; unpaired and paired image-to-image variants (e.g., CycleGAN/pix2pix–style formulations) are commonly adopted for domain adaptation between greenhouse and field imagery, reducing feature mismatches that derail correspondence search [150]. Recent agri-forestry research incorporates a modified unsupervised GAN directly into a dual-layer photogrammetry fusion stage to synchronize multi-altitude point clouds, enhancing the completeness of tree canopies in semi-arid environments [151]. Surveys on deep reconstruction also show that adversarial losses work well as priors for sharper textures and less “rubbery” depth around fine boundaries. This helps TSDF/Poisson fusion and NeRF/3D Gaussian Splatting surface extraction [152]. There are still practical concerns, such as mode collapse and hallucinations that do not fit the geometry, so practitioners usually link adversarial training to geometric constraints (such as multi-view photometric consistency, epipolar checks, or depth gradient losses) and use GAN outputs as inputs to classical or neural geometry instead of final geometry [153].

### 2.4. Learning-Based Methods

Recent advances in deep learning have made it possible to build three-dimensional (3D) geometry directly from two-dimensional (2D) images without having to match features or triangulate [154,155]. These learning-based methods use neural function approximation instead of explicit geometric modeling. In these methods, neural networks learn about scene structure, material properties, and lighting without being told to do so. Deep neural models can figure out depth, shape, and appearance from large training datasets, which makes 3D reconstruction possible even in difficult environments. This is different from traditional pipelines, which rely on handcrafted features [156].

#### 2.4.1. Neural Implicit Representations

Neural implicit representations encode three-dimensional shapes as continuous mathematical functions rather than discrete voxels or meshes, facilitating reconstructions at any spatial resolution [157]. In these models, a neural network takes a 3D coordinate f{x} = (x, y, z) as input and outputs a scalar value that shows a physical property, such as the probability of occupancy or the signed distance from a surface. The main benefit is that the network learns to show surfaces as level sets of this continuous function. This gets rid of the extra memory that dense voxel grids need [158]. Training usually means reducing the difference between predicted and real surface values from 3D datasets or multi-view observations. When the network is trained, it can make detailed meshes by marching cubes on the zero-level isosurface. Neural implicit surfaces are especially good for modeling plant organs with thin structures, such as leaves and stems, in agriculture. Voxel-based methods would need too much resolution for this [159].

**DeepSDF**: Deep Signed Distance Functions (DeepSDF) learn a way to connect spatial coordinates to their signed distances to the nearest surface to model 3D shapes [160]. The network gives positive distances for points that are outside the surface, negative distances for points that are inside, and zero distances for points that are exactly on the surface. The model learns to get close to this implicit surface during training by making the difference between predicted and ground-truth distances as small as possible. The signed distance field (SDF) encodes smooth spatial gradients, so the model can accurately fill in gaps between sparse samples. This makes it perfect for finishing partial or noisy reconstructions [161]. Because the SDF manifold is smooth, you can use differentiable rendering techniques to get a high-quality mesh. DeepSDF can recover parts of plants that are blocked or missing, such as inner canopy structures or fruits that are only partially visible, from limited views [162]. This is a precise but memory-efficient way to build things back up.

**Occupancy Networks**: Occupancy Networks [163] implicitly depict surfaces as the decision boundary of a binary classifier. The network predicts the likelihood that a specific 3D point is occupied by the object, represented as a value between 0 and 1, rather than distances. During training, 3D coordinates are taken from a bounding volume, and the binary cross-entropy between predicted occupancies and ground-truth labels from reference meshes is minimized. After training, the surface is rebuilt by running the network on a dense grid and taking out the 0.5-level isosurface. Occupancy networks are strong against noisy or incomplete data because they are based on probabilities [164]. They create watertight, high-resolution surfaces that are more accurate and memory-efficient than traditional voxel carving. In farming, they are good for putting together small organs such as seeds or fruits, where it is very important to be able to clearly define the boundaries for shape and volume analysis [165].

#### 2.4.2. Neural Radiance Fields (NeRF)

**Figure 9:**
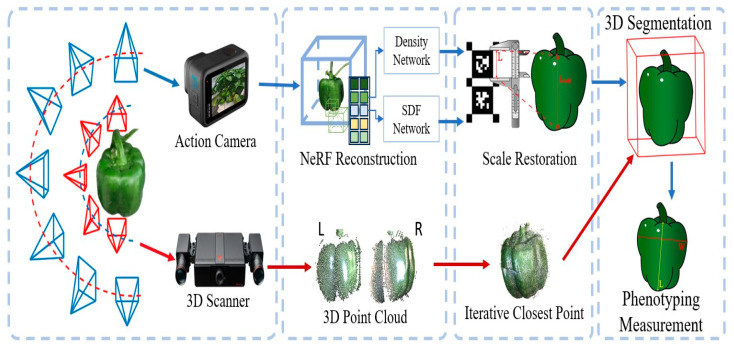
Framework for 3D phenotyping of bell pepper utilizing NeRF-based reconstruction, which integrates action camera and 3D scanner data for scale restoration and automated trait measurement [7].

The Neural Radiance Field (NeRF) model transformed 3D reconstruction by establishing a framework that directly acquires volumetric scene representation from multi-view RGB images. NeRF establishes a 5D continuous function F: (x, d) → (c, **σ**), wherein x = (x, y, z) represents a 3D position and d = (**θ**, ϕ) denotes a viewing direction. The output color c = (r, g, b) and volume density **σ** together tell us how light is emitted and absorbed at every point in space [166]. Using differentiable volume rendering equations, these values are combined along camera rays to create the image. NeRF adjusts its parameters during training to reduce the difference in brightness between rendered and real images [167]. This lets the network learn both geometry and appearance without being told to do so. NeRF can create photorealistic new views, but it usually needs dozens of views and static lighting. In farming, NeRF has been used to rebuild field crops and orchard canopies with sub-centimeter accuracy. This makes it possible to create continuous models that can be used for virtual inspection and canopy analysis [168,169]. Even though the basic NeRF model is strong, it has serious drawbacks in agricultural environments, especially when it comes to background clutter, dynamic outdoor lighting, and slow inference speeds. To overcome these difficulties, several domain-specific adaptations have been created. The framework for 3D phenotyping of bell pepper, which integrates action camera and 3D scanner data for scale restoration, is displayed in Figure 9.

**Plant-NeRF**: Plant-NeRF solves the main problem with regular NeRFs, which is that they cannot handle the changing light conditions that are common in outdoor farming. Plant-NeRF uses a voxel-based volume rendering method with special lighting regularization terms to separate scene geometry from environmental lighting effects. This makes it possible to reconstruct scenes accurately in natural light [170]. This method uses a shadow-masking system to get rid of artifacts that happen when shadows move or when things block each other, which is common in dense crop canopies. 1 Experimental validation on maize and cotton datasets demonstrated that Plant-NeRF achieves superior geometric accuracy compared to vanilla NeRF, reporting a determination coefficient (R^2^) of 0.993 for plant height and 0.988 for leaf width estimation, making it a robust tool for field-based phenotyping.

**Figure 10:**
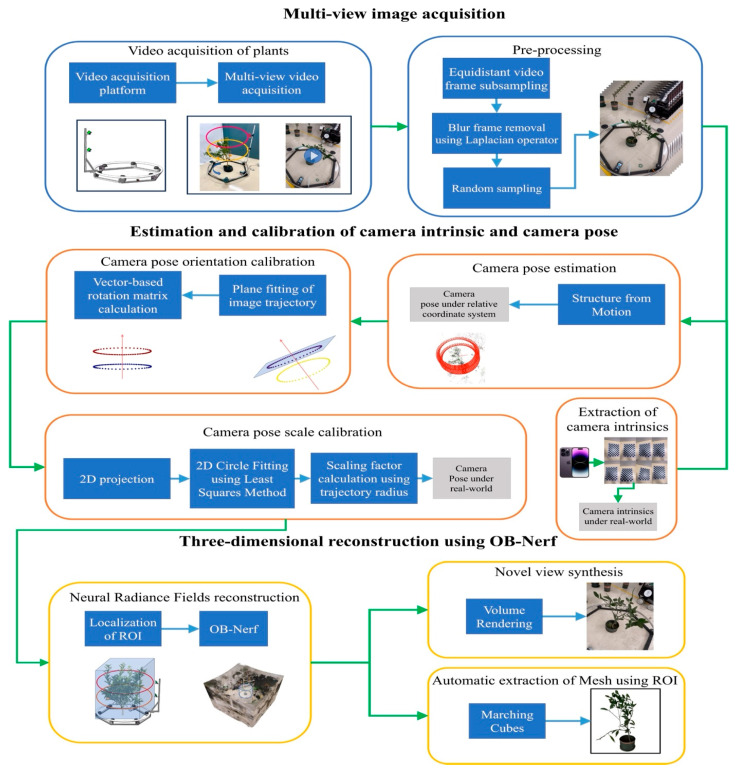
The OB-NeRF reconstruction pipeline, featuring an object-centric paradigm that localizes the Region of Interest (ROI) to separate complex canopy structures from background noise [45].

**OB-NeRF (Object-Based NeRF)**: OB-NeRF presents an object-centric reconstruction paradigm aimed at isolating the target plant from intricate, cluttered agricultural environments, minimizing the need for extensive manual annotation. This method uses a new ray sampling strategy that focuses on the “Region of Interest” (ROI) that contains the plant. This makes the calculations much faster and more accurate [171]. OB-NeRF cuts down the time it takes to reconstruct a single plant from hours to about 30 s by using a shallow Multi-Layer Perceptron (MLP) and multi-resolution hash encoding. This is a big step forward for high-throughput applications OB-NeRF also has an exposure adjustment phase to deal with uneven lighting and an automated pose calibration step. For leaf width, it has a Mean Absolute Error (MAE) of only 0.12 cm, which is better than Instant-NGP in terms of fine-scale structural fidelity. As demonstrated in Figure 10, this object-centric pipeline explicitly localizes the plant ‘Region of Interest’ to separate complex canopy structures from background noise.

**Mip-NeRF and Instant-NGP**: There are now several versions of NeRF that aim to address its issues with aliasing and computational requirements. Mip-NeRF reduces aliasing by using cone tracing instead of ray sampling. In this method, each pixel ray is modeled as a finite conical frustum instead of an infinitesimal line. This method lets you render at multiple scales and keeps fine details better when training with images taken at different resolutions.

Instant-NGP (Instant Neural Graphics Primitives) [172] presents a multi-resolution hash encoding that expedites NeRF training from hours to seconds. Instant-NGP does not require a lot of space to store voxel features. Instead, it maps 3D coordinates into small hash tables at different resolutions, which significantly reduces memory and processing needs. This new technology enables the reconstruction of objects almost in real-time on consumer-grade GPUs. These methods are revolutionizing agricultural phenotyping by enabling researchers to capture and reconstruct crop scenes in just a few minutes. This makes it easier to quickly extract traits and monitor growth.

**Recent NeRF Extensions**

They address the challenges of sparse-view inputs, dynamic scenes, and large-scale environments.

**FreeNeRF** introduces frequency regularization and adaptive sampling to enhance stability when there are limited input images. This maintains consistency in the structure, even when there is limited data [78].

**D-NeRF** (Dynamic NeRF) introduces a time dimension t to the radiance function, enabling learned deformation fields to reconstruct non-rigid and moving scenes [78]. This is especially important for farming, where plants can move because of wind or growth, which can cause changes over time.

**Block-NeRF** enables NeRF to operate in vast spaces by dividing the scene into “blocks” of space, training each one separately, and then seamlessly combining them for continuous rendering. This hierarchical structure enables the creation of large reconstructions of orchards or fields that would be too extensive for a single model to handle. These different versions of NeRF make it more suitable that it can be used in real-world agricultural situations, from studying one plant to rebuilding an entire field.

**Multispectral-NeRF** modifies the NeRF framework to process data collected from multiple spectral bands, in addition to the standard RGB [164]. Instead of three-color channels, each input image gives reflectance measurements across six or more wavelengths, which are usually in the visible and near-infrared ranges. The neural architecture is enhanced to support higher-dimensional spectral vectors, and the rendering function is adjusted to forecast per-band radiance values. Also, radiometric calibration makes sure that reflectance is linear, and input normalization keeps things the same across wavelengths. This method enables the simultaneous reconstruction of both the geometry and physiological characteristics of a 3D object. These include chlorophyll content, water stress, and nutrient distribution. Tests have shown that Multispectral-NeRF is more accurate in terms of spectral accuracy and depth consistency than standard NeRF and Instant-NGP. This makes it an important framework for multi-sensor fusion in precision agriculture.

**Zip-NeRF** is a hybrid radiance-field method that combines the speed of grid-based encodings with the anti-aliased rendering of cone-traced NeRF. This enables faster training and inference, eliminating moire, “floaters,” and shimmer on thin, high-frequency structures such as leaves and stems. Specifically, scene features are kept in multi-resolution grids and rendered with integrated ray footprints (cones/frusta) instead of point samples. This way, the detail stays the same when you zoom in or out and across different image resolutions. This is a problem that often happens with field captures when the optics and UAV heights change. Training follows standard posed-image volumetric losses, but its sampling/representation is optimized so convergence and FPS approach Instant-NGP while retaining Mip-NeRF’s robustness to scale changes. In agriculture, this means cleaner canopy boundaries, more reliable depth around foliage that blocks light, and easier reconstructions of large plots on standard GPUs. Depth maps from the field can be exported and meshed using Truncated Signed Distance Fusion (TSDF)/Poisson for trait computation (height, volume, LAI proxies) or combined with semantics for downstream phenotyping [8,127,173].

**Triplane Representations**

Triplane representations provide an effective balance between comprehensive volumetric grids and point-based encoding [78]. The method breaks down a 3D scene into three orthogonal 2D feature planes: XY, YZ, and XZ. Each plane holds learned latent codes that describe the scene’s local structure and appearance. During rendering, feature vectors from these planes are combined to figure out the color and density at any 3D point. This factorization significantly reduces memory usage while maintaining high-quality reconstruction. Triplane methods accelerate training and inference, making them a suitable choice for phenotyping setups with limited resources. However, performance may decline in scenes characterized by significant depth variation or intricate occlusions, as each triplane inherently presumes smooth variation along orthogonal axes.

**Multi-Plane Images (MPI)**

The Multi-Plane Image (MPI) representation is an early neural view-synthesis method that depicts scenes as stacks of semi-transparent image layers at specific depth intervals. Every layer has information about color and opacity. This allows you to create new views by alpha-compositing these planes based on the camera’s geometry. By minimizing photometric reconstruction loss, MPIs can be trained directly from stereo pairs or short image sequences. They render quickly and use little memory, but they struggle with scenes that feature strong depth discontinuities or significant changes in viewpoint. In agricultural imaging, MPI-based models can quickly display canopy structures or fruit clusters when sufficient computing power is not available. However, their geometric accuracy is not as good as that of modern implicit or radiance-field methods.

#### 2.4.3. Explicit Radiance

This category includes methods that prioritize real-time rendering and efficient surface extraction.

**Figure 11:**
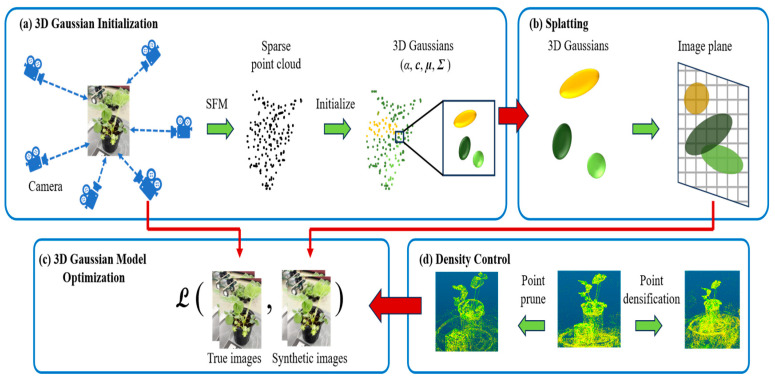
Framework of 3D Gaussian Splatting, detailing the four main stages: (**a**) 3D Gaussian Initialization, where multi-view images are processed via Structure-from-Motion (SfM) to extract a sparse point cloud that initializes the 3D Gaussian parameters; (**b**) Splatting, which projects the 3D Gaussians onto a 2D image plane to render the scene; (**c**) 3D Gaussian Model Optimization, where a loss function iteratively refines the model by comparing true captured images with the rendered synthetic images; and (**d**) Density Control, an adaptive process involving point pruning and densification to accurately capture intricate geometric details of the plant [129].

**3D Gaussian Splatting**: 3D Gaussian Splatting uses thousands to millions of anisotropic 3D Gaussians to represent a scene. The centers, covariances, opacities, and spherical-harmonic colors of these Gaussians are learned from start to finish [62]. The rendering process projects each Gaussian onto a screen-space ellipse and alpha-composites them, allowing for real-time novel-view synthesis with training times that are significantly shorter than those of NeRF, yet maintaining similar visual fidelity [62]. When compared to ray-marched radiance fields, 3D Gaussian Splatting usually converges in minutes, runs interactively, and has good memory/runtime trade-offs. This is especially true when it is used with densification/pruning schedules and mild geometric priors (such as depth/normal regularizers) to stop “floaters” [19,78]. In agriculture, tailored pipelines such as Wheat3DGS and Cotton3DGaussians, as well as plant-centric frameworks such as PSGR, demonstrate high-fidelity reconstructions for canopy/ear mapping, boll counting, and trait extraction in outdoor lighting conditions. This proves that 3D Gaussian Splatting is a fast, field-ready alternative to heavier neural volumetrics [17,57,163]. In practice, one starts with SfM poses, trains with photometric reprojection and sparsity/opacity regularization, and when watertight geometry is needed, exports a mesh via Poisson/TSDF from the learned Gaussian set [20,62,78]. The complete framework applied to plant phenotyping from Structure-from-Motion initialization to adaptive density control of the 3D Gaussians is detailed in Figure 11.

**4DGS**: 4DGS takes 3D Gaussian Splatting to the next level by adding a time-conditioned deformation field to each anisotropic Gaussian. The parameters (center, covariance, opacity, SH color) are set in a canonical space and then warped to any timestamp using learned per-Gaussian motion. Finally, the images are rasterized using visibility-aware alpha compositing [89]. Training enhances photometric reprojection across multi-view video frames, incorporating temporal smoothness and sparsity regularization. This results in temporally coherent, real-time novel-view rendering of non-rigid motion (e.g., swaying leaves, moving platforms) while maintaining the speed–quality trade-offs that rendered static 3D Gaussian Splatting appealing [62,145].

A practical pipeline starts a static 3D Gaussian Splatting at a key frame from SfM/poses and then fits both deformation and appearance over time. For measurement, time-sliced meshes or trajectories can be exported via TSDF/Poisson on the deformed Gaussians [78]. In agriculture, 4DGS naturally goes along with plant-focused 3D Gaussian Splatting toolchains for field phenotyping under wind and robotic operations. This makes it possible to quickly and accurately reconstruct models that can handle motion [19,78,145].

**Neuralangelo**: Neuralangelo reconstructs high-fidelity, watertight surfaces from posed video by optimizing a neural signed-distance field (SDF) that is hash-grid encoded for speed and detail, then extracting a mesh via marching cubes and baking view-consistent textures [76,94]. Neuralangelo’s differentiable surface rendering with numerical/finite-difference normals directly supervises geometry, capturing sharp edges, shallow relief, and specular materials that often defeat vanilla NeRF [76,173]. This is different from pure radiance-field methods that focus on view synthesis. The pipeline works: use SfM to guess where the camera is, train the SDF with photometric and normal losses in a coarse-to-fine schedule, extract/UV-unwrap the mesh, and optionally post-smooth or fuse with TSDF for scale alignment [100,127]. For agricultural “digital twins,” this creates metrically stable, mesh-native models of stems, trellises, machinery, and building interiors that work well with CAD/robotics stacks and trait computation downstream [69,76].

#### 2.4.4. Few-Shot and Single-View Reconstruction

These models are made to figure out 3D structure from just one 2D image, making them a great choice when you do not have multi-view data [159]. Explicit representations, such as 3D Gaussian Splatting, provide better rendering speed and quality, but to converge without artifacts, they typically require dense multi-view acquisition, which frequently requires 50–100 overlapping images. However, obtaining such dense inputs is either impractical or impossible in many real-world agricultural scenarios, such as robotic grasping, rapid field scouting, or historical dataset analysis. As a result, a unique class of “few-shot” and single-view techniques has been developed to infer 3D geometry in situations where data is limited.

**MiDaS**: MiDaS predicts relative depth from a single RGB image. You can then anchor it metrically with a scale prior (such as a known height or marker) and lift it to a point cloud and watertight mesh (such as Poisson) to compute traits. Newer DPT-backbone variants facilitate the generalization across datasets and recovery of fine details, which is crucial for thin leaves and twigs. In phenotyping rigs with limited resources, MiDaS pipelines are appealing because they do not require per-scene optimization or camera calibration, but they still provide useful geometry for biomass proxies. A recent study found sub-centimeter concordance with photogrammetry (RMSE ≈ 0.775 cm) when integrating MiDaS depth with Poisson, which is beneficial for economical plant scanners, provided meticulous scale calibration and unobstructed backgrounds.

**Monocular Diffusion + Distillation**: These techniques create new views from a single image using a conditional diffusion model, then bend those views back to 3D (volumetric occupancy), and finally turn the expensive pipeline into a fast feed-forward network [74]. Depth-distillation from strong monocular predictors improves the depth/geometry consistency, which reduces artifacts in areas with low texture or foliage [74]. Previous research on single-image diffusion-to-3D demonstrated the efficacy of 2D priors in generating absent views for reconstruction [89]. Results from driving datasets indicate effective transferability to field robotics, where only a single camera shot may be accessible per plant/row [74].

**3D-PRNet for Counting Objects**: 3D-PRNet uses a sparse 3D set of object centers to estimate counts from a single RGB image. It does this by regressing the centers using a FoldingNet-style latent prior and permutation-invariant set losses. In scenes that are tightly packed and blocked, it reports about 98% accuracy [75]. This single-image, sparse-3D method is useful for agriculture as a yield proxy for fruit bins or dense clusters when capturing multiple views or using depth sensors is not possible. It is also much better at dealing with foliage occlusion than 2D detectors. You can reduce the remaining scale ambiguity by anchoring to a known dimension or getting cues from strong monocular depth predictors such as DPT/MiDaS [70,73].

**DreamGaussian**: DreamGaussian converts a single RGB (or text prompt) into a 3D scene by optimizing a cloud of anisotropic 3D Gaussians each with position, covariance, opacity, and SH color using score-distillation sampling (SDS) from a pretrained 2D diffusion model: the Gaussians are differentiably rasterized to novel views, the diffusion prior supplies a gradient toward more plausible renderings, and the Gaussian parameters are updated to “hallucinate” unseen views and close occlusions [77,157]. Because Gaussians render analytically on the GPU (no per-ray MLP), training and playback are fast and interactive compared with NeRF-style volumetric fields, while a Gaussian-to-mesh step with UV-space texture repair yields exportable, watertight assets for downstream use. Practical recipes use light regularizers such as foreground masks, monocular depth/normal priors, and sparsity to stabilize thin structures (such as leaves and tendrils) and get rid of small “floater” artifacts that can happen when you only use text and images. DreamGaussian is useful for quickly making low capture “digital twins” from one or a few images in agricultural workflows. For metric traits, users usually use a global scale (ruler or marker) and, if necessary, combine the result with geometric cues (SfM/TSDF) to reduce scale ambiguity and improve fidelity in dense canopies [68,78]. Recent field studies utilizing 3D Gaussian Splatting variants corroborate this trajectory, indicating high-fidelity crop reconstructions and phenotyping from sparse imagery. This suggests that DreamGaussian-style pipelines can integrate into rapid scouting and visualization, even when comprehensive multi-view capture is unfeasible [78].

**PixelSplat**: PixelSplat takes one (or a few) RGB images and turns them into a set of 3D Gaussians in one pass. A pretrained visual encoder first makes dense per-pixel features. Then, a multi-view epipolar transformer combines feature evidence along epipolar lines to find Gaussian centers and make a joint prediction of a global scale factor. This solves the classic monocular scale ambiguity problem without needing external calibration [79]. Heads regress each Gaussian’s covariance, opacity, and spherical-harmonic color. A differentiable 3D Gaussian Splatting renderer supervises training using photometric reprojection and simple priors (such as alpha/sparsity/normal cues), so there is no need to optimize each scene separately [62,79]. In practice, this gives you stable geometry from just a few views while still allowing for real-time rendering, which is a big advantage over NeRF-style ray marching [62,78]. PixelSplat’s encoder can use strong foundation features such as DINOv2 to make predictions about traits such as height and volume more accurate from limited images [79,80]. For agriculture, it fits right in with fast scouting and phenotyping workflows that are already using Gaussian-based reconstructions. This makes it easy to see the canopy quickly or map instances from sparse captures [66,67].

**Figure 12:**
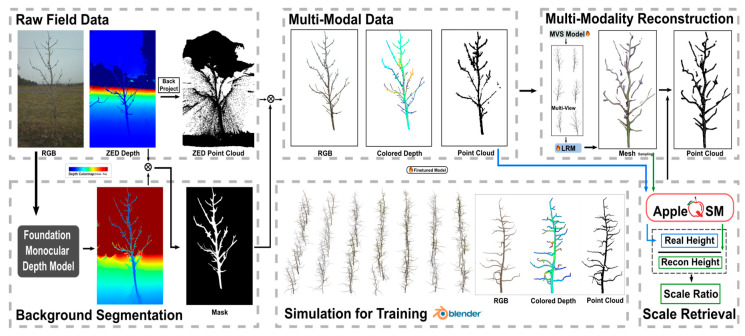
Overview of the DART (Diffusion-based Apple Tree Reconstruction) framework, showing the processing of multi-modal field data through background segmentation and LRM regression to generate 3D representations [80].

**DATR** (Diffusion-Based Apple Tree Reconstruction) is a hybrid learning pipeline that works with sparse views and is designed for whole-tree modeling when there are obstructions and clutter. It first uses a learned segmenter to remove the sky, ground, and background from a few RGB views of a single tree. Then, it uses on-board depth to anchor scale and recovers camera intrinsics and poses with a standard SfM step (masking reduces mismatches on foliage) [81]. After that, a conditional diffusion module imagines missing structure and combines cues from each view into a rough multi-view point cloud. A transformer called the “Large Reconstruction Model” (LRM) then turns this into a mesh that is consistent with topology and keeps thin branches and branch continuity [81]. Final meshing employs volumetric fusion techniques (e.g., TSDF/Poisson) to produce a watertight, textured tree model appropriate for trait analysis and simulation. DATR, which was trained on synthetic apple-tree data and tested on real orchards, shows better branch-level completeness than classical MVS/SfM baselines in the sparse-view regime while still being useful for field phenotyping [42,81]. Common failure modes are motion caused by wind, depth noise on shiny leaves, and scale drift when there are no reliable priors. In these situations, cross-scene plant pipelines (such as PSGR/P3DFusion) or Gaussian-splatting back-ends can help make the system more stable [51,52]. Figure 12 provides an overview of this framework applied to apple trees, showing how multi-modal field data is processed to extract precise height and scale metrics.

#### 2.4.5. Feed-Forward and Foundation Models Without Per-Scene Optimization

**VGGT, DUSt3R and MASt3R**: is a large, feed-forward transformer that takes in anywhere from a few to hundreds of uncalibrated images and predicts camera intrinsics/extrinsics, dense depth/normal maps, per-view pointmaps, and a fused point cloud in one pass. This is usually done without any per-scene optimization, which allows for sub-second end-to-end inference on modern GPUs [19]. Training combines transformer features with geometry-aware losses (photometric/reprojection/correspondence) across a variety of real and synthetic datasets, resulting in strong generalization to broad baselines and mixed focal lengths [3,19]. In ag pipelines, VGGT can either start SfM or act as a direct metric reconstructor when a simple scale prior is available (e.g., stake height, UAV altitude, or IMU). After that, NeRF/3D Gaussian Splatting back-ends can improve texture and fill in thin foliage [32,82]. Common failure modes are blur caused by the wind and very repetitive canopy textures. To fix these problems, you can use short exposure, slight capture parallax, and lightweight pose/scale smoothing [19,83].

**MapAnything**: MapAnything is a unified, feed-forward transformer that can do more than a dozen 3D vision tasks with just one backbone and lightweight heads. It takes in image sets and optional geometry (such as depth maps, known camera poses, or scale priors) and directly regresses metric outputs, such as camera intrinsics/extrinsics, dense depth/normals, per-view pointmaps, and a fused point cloud, in a single pass without having to optimize each scene [83]. The design employs cross-view attention and geometry-aware supervision, enabling tasks to co-train one another, resulting in robust generalization to extensive baselines and varied focal lengths characteristic of field capture [3,83]. In agricultural pipelines, MapAnything can use handheld or UAV images to create full reconstructions. It can then pass these on to TSDF, NeRF, or 3D Gaussian Splatting modules for texturing and thin-structure refinement. When absolute scale is important, a simple prior (stake height, GPS/IMU altitude) makes outputs immediately metric [42,84]. MapAnything’s multi-task formulation reduces failure on sparse or weak-texture scenes compared to other feed-forward geometry models (e.g., VGGT, DUSt3R/MASt3R), but it still benefits from short exposures and small capture parallax to limit wind blur and aliasing [19,44,45].

**PETR and BEVFormer:** Both methods take 2D image features and put them into a metric Bird’s-Eye-View (BEV) grid. This lets downstream heads do 3D reasoning directly in top-down space, which is great for navigation, mapping, and object layout [85,86]. PETR does this by changing camera-aware position embeddings and depth-aware queries so that per-view features are projected onto a common BEV plane. This lets end-to-end detection work with either a single camera or multiple cameras [83]. BEVFormer builds on this idea by using spatiotemporal transformers and deformable attention to combine evidence from multiple frames and views into a single, long-lasting BEV memory. This makes the system more stable when the lighting changes or the object moves [85]. The same BEV map can be used in agriculture to show crop-row segmentation, fruit/obstacle detection, and traversability layers for ground robots or UAVs. In real-world situations, scale is set with GPS/IMU or SfM calibration, and LiDAR depth may be added to stabilize geometry in cluttered canopies [14,42,84]. Recent greenhouse and field robots demonstrate that BEV-centric perception seamlessly integrates with semantic SLAM and path planning, indicating that BEV transformers are well-suited for row-structured farms and orchards [87].

**GAMBA** works with single-image 3D by treating a set of learnable 3D-Gaussian tokens as a long sequence that is processed with a Mamba state-space backbone. It uses image features and camera embeddings to predict each primitive’s center, anisotropic covariance/scale, opacity, and color. These predictions are then rendered differentiably using 3D Gaussian splatting [43,88]. Training reduces photometric loss between splat renderings and the input (including synthesized or augmented views) using sparsity/opacity regularizers to prevent floaters. Cross-attention between tokens and CNN/ViT features adds semantics from the single RGB [88]. Single-view reconstruction has scale ambiguity, so GAMBA uses known intrinsics/markers or monocular priors to anchor scale, and it also uses techniques from PixelSplat and DreamGaussian to stabilize Gaussians when there are not many views [77,79]. The outcome is rapid, feed-forward inference that produces a directly renderable 3D Gaussian scene useful for field snapshots where only one image per plant/row is possible and easily integrated with subsequent meshing or trait extraction in agricultural pipelines.

### 2.5. Comparative Analysis of Reconstruction Methods

**Figure 13:**
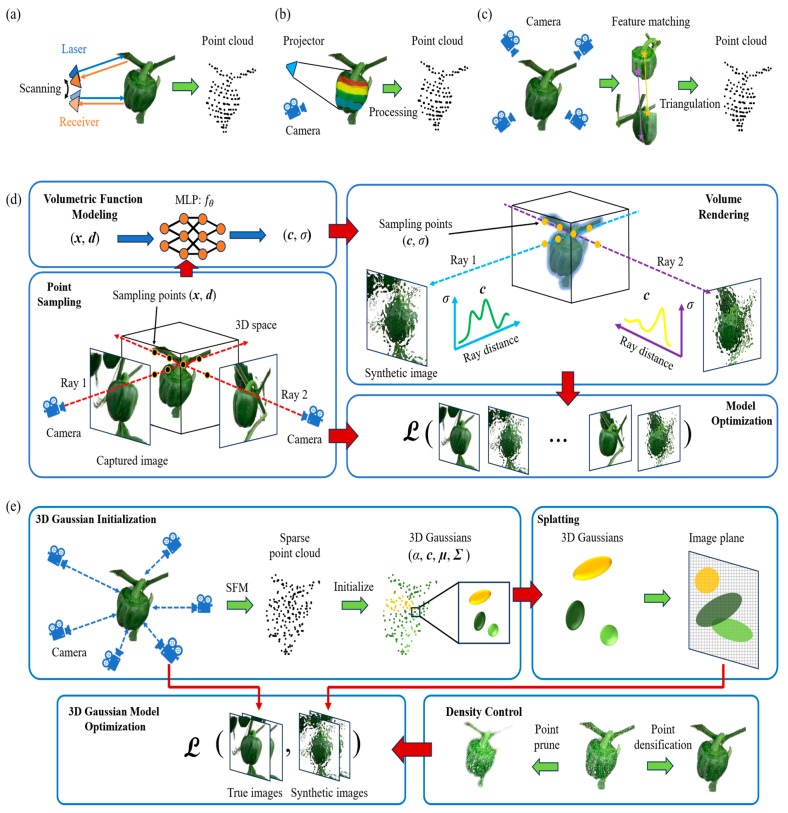
Comparative analysis of 3D reconstruction methodologies applied to a single specimen to highlight differences in output density and geometric fidelity. The specific pipelines depicted are: (**a**) LiDAR, which uses laser scanning to generate a point cloud; (**b**) Structured Light, which projects patterns onto the object to recover surface geometry; (**c**) Structure-from-Motion (SfM), which relies on multi-view feature matching and triangulation; (**d**) Neural Radiance Fields (NeRF), showing volumetric function modeling, point sampling along rays, and volume rendering; and (**e**) 3D Gaussian Splatting, detailing its initialization from a sparse point cloud, splatting projection, model optimization, and density control [7].

3D reconstruction has grown from a few specialized methods to a wide range of methods. From classical photogrammetry to active sensing such as LiDAR to the groundbreaking idea of neural rendering, each method has its own pros and cons in terms of accuracy, cost, speed, and visual fidelity. This analysis brings together results from many fields, such as architecture, robotics, agriculture, materials science, and underwater imaging, to create a clear framework for comparison. Structure-from-Motion (SfM) and Multi-View Stereo (MVS) are examples of classical geometry-based methods that are still the most important for many applications, especially in plant phenotyping and architecture. They provide inexpensive, understandable pipelines that can get millimeter-level accuracy with cameras that are meant for consumers. But they often take a lot of processing power and have trouble with surfaces that do not have texture, complex occlusions, and changing environments. A direct visual comparison of these techniques applied to a single fruit specimen is presented in Figure 13, highlighting the differences in output density between LiDAR, structured light, SfM, NeRF, and 3D Gaussian Splatting.

Simultaneous Localization and Mapping (SLAM), whether it is visual (V-SLAM) or LiDAR-based, focuses on estimating the trajectory in real time for autonomous navigation. ORB-SLAM3 and LiDAR-Inertial SLAM (such as LIO-SAM and FAST-LIO2) are examples of systems that have centimeter-level pose accuracy and are used as benchmarks for robotics. Their main output is a trajectory, but the maps they make, which can be anything from sparse point clouds to dense geometric models, are being combined with other methods more and more.

Deep learning has brought about a major change in the way things work. Neural Radiance Fields (NeRF) set a new high bar for photorealism by showing scenes as continuous implicit functions, but this came at the cost of long training and rendering times. 3D Gaussian Splatting came along later and broke this trade-off by using an explicit, differentiable representation to give both the best visual quality and the fastest rendering speeds. These neural methods are great at capturing how things look and dealing with complicated, view-dependent effects. This makes them perfect for digital twins and virtual reality.

There is a clear trend toward hybrid systems in all these areas. Geometric methods give neural models strong starting poses, LiDAR data gives vision-based pipelines ground-truth scale and density, and semantic segmentation from deep learning models helps improve and understand reconstructions. The table below shows a detailed comparison of these methods, including their main metrics and the pros and cons of each. Table 1 provides a qualitative comparison of various 3D reconstruction methodologies, detailing their core principles, strengths, limitations, and primary application domains.

A thorough look at 3D reconstruction and sensing technologies shows that the field is changing quickly and across many domains, going from traditional geometric pipelines to hybrid, learning-driven, and explicit neural representations. A synthesis of performance across autonomous navigation, neural rendering, agriculture, and materials science reveals distinct methodological tiers and domain-specific benchmarks. In robotics and autonomous navigation, multi-sensor fusion SLAM is the only standard. Surveys on 3D LiDAR SLAM show that tightly coupled hybrid frameworks, especially LiDAR-Inertial Odometry (LIO) systems such as Fast-LIO2 and R3LIVE, can estimate poses with an Absolute Trajectory Error (ATE) of less than 1 cm and can work in real time at speeds of more than 100 Hz. Hybrid Visual SLAM methods such as Differentiable Recurrent Optimization-Inspired Design—Simultaneous Localization and Mapping (DROID-SLAM) set the standard for visual navigation, with ATEs below 2 cm. Specialized agricultural UAV applications, which must deal with low-overlap (35–45%) and repetitive textures, now use custom algorithms such as LORP-Match (Low-Overlap Repetitive Pattern-Match) to achieve real-time (25 FPS) pose reconstruction with RMSE of 1.7 cm, which is better than traditional SfM and generic V-SLAM methods.

3D Gaussian Splatting has clearly taken over for Neural Radiance Fields (NeRF) in the fields of vision-based reconstruction and neural rendering. Several surveys show that 3D Gaussian Splatting strikes the best balance between quality and performance, with real-time rendering (150–250 FPS) and quick training (minutes), as well as setting SOTA visual fidelity standards (PSNR 34–36 dB, SSIM ~0.97, LPIPS < 0.03). This superiority extends to sparse-view and single-view reconstruction, where 3D Gaussian Splatting and new Transformer/diffusion-based models (such as HoloRecon) show the best performance, with single-view PSNRs of up to 34.2 dB.

Plant phenotyping is the field that has made the most progress in terms of new and improved methods. While inexpensive classical pipelines such as 3DPhenoMVS (using DSLR) and smartphone-video SfM (using COLMAP) work very well, with R^2^ > 0.97 and RMSE as low as 2.8 mm compared to LiDAR ground truth, specialized neural models work better for certain tasks. OB-NeRF is much better than standard NeRF and 3D Gaussian Splatting for complex, hidden canopies. It has a PSNR of 35.7 dB and an RMSE of 0.98 mm. For dynamic (non-rigid) plants, a multi-sensor fusion approach (RGB + Depth + LiDAR + NICP) creates a new Temporal Stability Index (TSI) metric (0.94) and gets sub-millimeter accuracy (RMSE 0.8–1.2 mm). A hybrid 2D-to-3D segmentation pipeline (DeepLabv3 + SfM) also works surprisingly well for semantic segmentation, with a mIoU of 84.7%, which is better than native 3D deep learning models such as PointNet++ (mIoU 78.6%).

Finally, specialized domains set the limits for performance. In materials science, X-ray CT reconstruction of asphalt sets a standard for ultimate geometric accuracy, with an RMSE of 0.005–0.009 mm. On the other hand, in difficult active sensing environments, 3D-UFSPI can reconstruct 3D images in water with a high turbidity (50 NTU) and low sampling rates (15%). Medical imaging pipelines show that classical geometry (SfM + MVS + Poisson) can produce high perceptual quality (PSNR 37.1 dB) even from compressed JPEG data. Hardware improvements are helping this convergence along. For example, new solid-state OPA LiDAR sensors promise to make real-time (over 200 Hz) active sensing accurate to within a centimeter and cheap (less than $300). Table 2 presents quantitative performance benchmarks for various 3D reconstruction methodologies, evaluating their key metrics and practical applications across different domains.

### 2.6. Field Deployment Compatibility

When implementing 3D reconstruction techniques in actual agricultural settings, it is important to strike a careful balance between hardware constraints, computational efficiency, and resilience to unpredictable field conditions such as wind, varying illumination, and canopy occlusions. In contrast to controlled laboratory environments, open-field deployment necessitates systems that can function with acceptable reconstruction fidelity under stringent latency constraints.

#### 2.6.1. Geometric Photogrammetry (SfM/MVS)

Since they can achieve millimeter-level accuracy with just consumer-grade cameras or smartphones, traditional Structure-from-Motion (SfM) and Multi-View Stereo (MVS) pipelines continue to be very appealing for low-cost deployment [29,37]. However, environmental sensitivity significantly restricts their practical field compatibility. Even slight wind-induced leaf motion results in ghosting artifacts, blurred geometry, or missing plant parts because these techniques assume static scenes. Dense canopies create significant canopy occlusion problems, where overlapping plant organs prevent cameras from capturing internal structures, leading to incomplete “hollow” 3D models [89]. Furthermore, performance deteriorates under varying illumination, particularly on reflective surfaces such as waxy leaves that create specular highlights under shifting natural light. These pipelines often run slowly, frequently requiring 20–30 min per model, which limits their usefulness for real-time decision-making [90].

#### 2.6.2. Active Sensing and SLAM

When deployed outdoors, active sensing techniques in particular, LiDAR and SLAM systems offer noticeably greater robustness. Absolute scale, robust resistance to lighting variation, and the capacity to penetrate canopy layers using near-infrared laser returns are all provided by LiDAR-based mapping, as the system provides its own light source and does not rely on ambient solar radiation. This ability to “see through” foliage layers allows for the mapping of biomass and structural traits both above and below the canopy, directly addressing canopy occlusions. While mobile (MLS) and aerial (ALS) systems trade some accuracy (1–15 cm) for scalability, terrestrial laser scanning (TLS) achieves incredibly high accuracy (1–10 mm) but is static and labor-intensive. The optimization of contemporary real-time SLAM systems for embedded deployment is growing. For example, on embedded UAV computers, Fast-LIO2 completes mapping iterations in less than 25 ms by directly registering raw LiDAR points without feature extraction. By closely integrating LiDAR, visual, and inertial measurements to produce RGB-colored dense maps, R3LIVE further enhances real-time performance. While LSD-SLAM offers semi-dense reconstructions in low-texture regions and is susceptible to motion blur, visual SLAM frameworks such as ORB-SLAM3 offer computational efficiency (≈29 ms tracking) but perform poorly in textureless scenes. Viewpoint invariance is improved by hybrid deep-feature techniques such as DROID-SLAM and LORP-Match, which have been effectively used for UAV-based agricultural monitoring in croplands and orchards to handle occluded regions [91]. Since sunlight overpowers infrared projection and reflective leaves deteriorate measurement quality, RGB-D and ToF cameras are still not very suitable for outdoor fields, despite their quick millimeter-level depth at close range.

#### 2.6.3. Rendering and Reconstruction of Neural Networks

**Neural Radiance Fields (NeRF)**: The advantages of NeRF include outstanding performance in handling intricate canopy occlusions and view-dependent effects by leveraging learned implicit priors to “fill in” internal structures and partially occluded plant organs. The disadvantage is that standard NeRF requires static scenes and constant lighting, and because training takes hours, it is not suitable for real-time field use. Specialized versions, such as Plant-NeRF, enhance compatibility by managing varying illumination through shadow-masking systems and lighting regularization to separate scene geometry from dynamic shadows and shifting natural lighting.

**3D Gaussian Splatting**: The advantages of 3D Gaussian Splatting are real-time rendering (>60 FPS) on consumer GPUs and speedy convergence in a matter of minutes [43]. Explicit frameworks such as OB-NeRF improve occlusion handling by focusing sampling on the plant’s “Region of Interest” to separate complex canopy structures from background noise [46]. Accurate SfM poses, and static scenes are still needed for initialization (very poor wind robustness).

**4D Gaussian Splatting (4DGS)**: The advantages of 4DGS are that it is specifically designed for dynamic scenes and adds a time-conditioned deformation field to handle non-rigid motion, such as swaying leaves [69]. The disadvantages include more complicated calibration and higher processing demands.

#### 2.6.4. Fast Feed-Forward 3D

**Methodology (MapAnything, DUSt3R, VGGT):** The advantages include sub-second, end-to-end inference without per-scene optimization, making them highly suitable for quick field scouting. They can withstand unposed or sparse inputs and may offer structural resilience against canopy occlusions by leveraging pre-training on large synthetic plant datasets to learn underlying branching rules. Still vulnerable to wind-induced blur and generally less accurate than optimization-based techniques (about 2–3% of LiDAR benchmarks). Open-field studies are not currently compatible. It is still the gold standard for micro-scale (micron-level) internal structure analysis of roots and seeds, but it requires bulky, costly, and stationary lab equipment.

### 2.7. Accuracy Metrics and Evaluation Criteria

This section discusses how to measure approaches in terms of quantities. Choosing the best 2D-to-3D method for the limitations of real-time farming conditions. The best choice is not the one that is best for everyone; it is the one that strikes the best balance between speed, cost, and accuracy.

This analysis will first establish a framework for accuracy validation to help distinguish between different approaches. Second, it will list the most important factors to consider when choosing a site for agriculture, with a focus on how well it can handle challenges in the field (such as wind, lighting, and occlusion) and its costs.

**Validation Framework: Perceptual vs. Geometric:** Neural rendering techniques (NeRF, 3D Gaussian Splatting) are usually tested by how good the images look from different angles (PSNR, SSIM, LPIPS). Classical photogrammetry (SfM/MVS) and LiDAR pipelines, on the other hand, are tested by their geometry (e.g., RMSE to ground truth). This difference is important for phenotyping because getting accurate measurements of traits such as height, LAI, and biomass requires both accurate geometry and renderings that look right. A lot of radiance-field papers focus on perceptual metrics and require a step to extract the surface for geometric comparisons, which can be wrong. To show that learning-based methods can be changed to consider geometry instead of just appearance, specialized, plant-oriented neural pipelines explicitly add geometric priors/losses and report trait-level validation (for example, strong R2 and low trait MAE).

**Geometric & structural fidelity metrics**: These metrics measure how physically accurate the rebuilt 3D model is compared to a ground-truth reference, which is usually a high-precision LiDAR scan, a high-accuracy photogrammetry model, or a destructive manual measurement. These are the most important numbers for agricultural phenotyping and navigation.

**RMSE (distance to ground truth)** and **R^2^**: Plant-NeRF and other learning-based methods report errors of less than 0.8 cm RMSE (<0.8 cm RMSE). The specialized OB-NeRF, which is made for complicated plant canopies, has an amazing 0.98 mm RMSE against ground truth and an R^2^ > 0.97 for trait extraction. This shows that specialized neural methods can match or even beat high-end geometric methods. For example, classical SfM + MVS (Metashape) has a 1.12 mm RMSE in studies that compare it to other methods. Even in tough, changing situations, a Non-Rigid Plant Fusion (RGB + Depth + LiDAR + NICP) pipeline can keep the RMSE between 0.8 and 1.2 mm.

Low-cost methods are also very effective, with Smartphone Video SfM getting 2.8 mm RMSE and an R^2^ of more than 0.99.

**Absolute Trajectory Error (ATE)**: LiDAR-Inertial SLAM systems demonstrate the highest precision. Fast-LIO2 and R3LIVE achieve ATEs of <1 cm. Visual SLAM (V-SLAM) is also highly capable, with DROID-SLAM achieving < 2 cm ATE and ORB-SLAM3 at 1.5–2.5 cm ATE.

**Semantic metrics (mIoU/BO)**: Recent research indicates that a Hybrid 2D-to-3D Segmentation method (e.g., DeepLabv3 + SfM) attains a cutting-edge 84.7% mIoU. This hybrid method, which projects high-quality 2D segmentation masks into a 3D point cloud, has been shown to work better than native 3D deep learning models such as PointNet++ (which got a score of 78.6% mIoU in the same study). This means that using the power of 2D foundation models (such as SAM, which we talked about in Section 2.4.2) is a better way to go than trying to segment large, noisy 3D point clouds directly.

**Photorealistic and Perceptual Fidelity Metrics:** These metrics, originating from image and video processing, quantify the visual similarity between a novel view rendered from the 3D model and a ground-truth photograph taken from that same viewpoint. They are the primary benchmarks for NeRF and 3D Gaussian Splatting.

**Peak Signal-to-Noise Ratio (PSNR):** Application & State-of-the-Art: The most common metric for novel-view synthesis quality. 3D Gaussian Splatting sets the state-of-the-art, averaging 34–36 dB. NeRF variants are close behind at 31–35 dB. Specialized models such as OB-NeRF (35.7 dB) and single-view HoloRecon (34.2 dB) confirm this high standard of photorealism.

**Structural Similarity Index Measure (SSIM):** Application & State-of-the-Art: Used alongside PSNR. 3D Gaussian Splatting achieves ~0.97 SSIM. NeRF achieves ~0.96 SSIM.

**Learned Perceptual Image Patch Similarity (LPIPS):** Application & State-of-the-Art: 3D Gaussian Splatting achieves a state-of-the-art LPIPS of <0.03. Table 3 presents a decision matrix for agricultural 3D reconstruction methods, comparing techniques across key parameters including geometric accuracy, perceptual fidelity, processing speed, cost, and environmental robustness.

## 3. Applications in Agriculture

3D reconstruction is useful in many ways, which gives it operational value. The shift from “images” to “models” makes it possible to get traits and automate tasks that were not possible before.

### 3.1. High-Throughput Phenotyping (HTP) and Morphological Characterization

High-Throughput Phenotyping (HTP) is the most advanced use of 3D reconstruction. Its goal is to measure plant architecture without damaging it. Modern breeding programs require examining thousands of genotypes, and the primary challenge in doing so manually is the time-consuming nature of the process.

Extracting Static Traits: Recent frameworks employing 3D Gaussian Splatting have achieved exceptional accuracy in extracting static traits. For example, the Cotton3DGaussians system reported a Mean Absolute Percentage Error (MAPE) of 2.38% for plant height and 3.66% for canopy width when compared to LiDAR ground truth. This accuracy also applies to phenotyping at the organ level. The Wheat3DGS pipeline has been successfully used in the field to separate and measure wheat heads. The per-instance MAPEs for length and width were 15.1% and 18.3%, respectively. These error rates for fine organs indicate that there is still room for improvement, but they represent a significant step forward in the automated counting of yield components that are typically counted by hand.

Seed and Organ Analysis: 3D reconstruction is revolutionizing the way we examine harvested products. A study on seed phenotyping in maize, wheat, and rice demonstrated that 3D Gaussian Splatting pipelines can accurately reconstruct seeds, yielding R2 values exceeding 0.93 for length and volume. This ability is crucial for evaluating the quality of seeds and identifying relationships between genotype and phenotype for traits that influence grain filling. 3D reconstruction captures the volumetric plumpness and surface rugosity of the seed, which are important signs of health and vigor. This is different from 2D scanners.

Standards for Validation: Increasingly, these phenotypic measurements are being verified against rigorous standards to ensure their accuracy. Scientists have made 3D-printed reference models, such as a fake sugar beet, to use as ground truth targets. These invariant models enable the accurate calibration of scanning systems. They show that consumer-grade 3D reconstruction can have errors as small as −10 mm to +5 mm when conditions are controlled. This type of standardization is crucial for comparing results from different HTP platforms and seasons.

### 3.2. Yield Estimation and “Digital Defoliation”

Accurate yield prediction is essential for supply chain management. 3D reconstruction offers a distinct advantage over 2D counting by addressing the “occlusion problem.” In a 2D image, a fruit hidden behind a leaf is invisible. In a 3D model generated from multiple viewpoints, it is possible to perform “digital defoliation” electronically removing the leaf layers to reveal the fruit clusters beneath.

In orchard crops, NeRF-based pipelines have been employed to estimate tomato yield with high fidelity. By reconstructing the full geometry of the plant, researchers achieved an R^2^ of 0.96 for fruit volume estimation, with a MAPE of 0.135. This approach allows for the volumetric assessment of yield, which correlates more strongly with biomass and market value than simple counts. Furthermore, the explicit nature of 3D Gaussian Splatting allows, for instance, segmentation in 3D space. In comparative studies, 3D Gaussian Splatting-based methods for fruit counting demonstrated superior Intersection over Union (IoU) and precision compared to FruitNeRF and standard MVS, largely due to the Gaussian representation’s ability to model the distinct boundaries of individual fruits in cluttered clusters.

### 3.3. Robotic Navigation, Manipulation, and Harvesting

For agricultural robots, the world is 3D. Navigation requires determining traversability, while harvesting requires precise hand-eye coordination.

**SLAM Framework:** SLAM applies the concepts of geometric reconstruction to real-time mapping [2]. Unlike offline methods such as SfM, SLAM continuously estimates both the camera’s trajectory and updates the 3D map while images are being captured. The system detects and tracks visual features across frames, and when equipped with an inertial measurement unit (IMU), utilizes optimization techniques such as Perspective-n-Point (PnP) or visual-inertial odometry (VIO) to minimize drift. This enables robots to navigate independently and rebuild plant environments on-the-fly. For instance, robots equipped with stereo cameras utilize visual SLAM to map orchards and determine canopy density for immediate decision-making [92].

**Semantic Mapping and Navigation:** 3D Gaussian Splatting-SLAM (Simultaneous Localization and Mapping) is becoming a powerful tool for creating dense, semantic maps of agricultural areas. 3D Gaussian Splatting maps show the environment in a continuous way, such as sparse feature maps. This lets robots see not only “obstacles,” but also specific types of objects, such as “trunk,” “row,” or “human.” This semantic awareness facilitates smarter path planning in orchards, where the canopy can block GPS signals.

**Collision Avoidance and Manipulation:** Robotic harvesting arms must navigate a maze of wires and branches to reach some fruit. New collision avoidance algorithms now use Gaussian models of both the robot and the environment. Planners can quickly determine the chances of a collision and create safe paths through the canopy by modeling the robot’s links and the plant’s branches as ellipsoids. The Fast Fruit 3D Detector (FF3D) framework is a good example of this integration. It utilizes a 3D CNN to detect objects and a next-best-view estimator to direct the robot’s camera to a position where it can view the object without obstruction, resulting in a grasping position error of less than 6.2 mm. Real-time 3D processing enables people to move around and see better, a unique ability.

### 3.4. Functional Modeling: Light Interception and Digital Twins

3D reconstruction serves as the geometric foundation for Functional-Structural Plant Models (FSPMs). Researchers can make “Digital Twins” that model physiological processes by bringing in high-quality 3D meshes of real plants into simulation environments.

**Light Interception Simulation:** The arrangement of leaves affects how well a crop can photosynthesize. Researchers use datasets such as MaizeField3D to perform ray-tracing simulations on reconstructed meshes, measuring how much light each organ can capture. These simulations have demonstrated that 3D models substantially mitigate the overestimation of canopy photosynthesis prevalent in 1D (layered) or 2D models, thereby offering a more precise foundation for yield prediction. When managing an orchard, 3D models of peach and plum trees enable growers to test how different pruning methods affect light penetration, which helps them plan the best way to shape the canopy before making a single cut.

**Disease Dynamics:** FFSPMs combined with 3D reconstructions are also used to predict how diseases will spread. Researchers have used these digital twins to model how splash droplets of fungal spores spread through a wheat canopy, connecting the plant’s specific structure to how likely it is to get sick. This provides a powerful method for breeding ideotypes that are resistant to disease based on their structure, rather than just their chemical immunity.

### 3.5. Multi-Modal Fusion and Stress Detection

Combining 3D geometry with spectral data (thermal, multispectral, hyperspectral) unlocks the ability to map physiological stress spatially.

**NIRSplat and Water Stress:** The NIRSplat framework combines 3D Gaussian Splatting with Near-Infrared (NIR) images. This fusion enables us to visualize drought stress in 3D because NIR reflectance is closely related to water content and cell structure. The 3D model allows you to separate plants from the soil, providing a clear indication of plant health. This is different from 2D NIR images, which combine the plant signal with the soil background.

**Salinity and Early Detection:** 3D reconstruction has been shown to be sensitive enough to detect changes in shape that occur in response to stress before they are visible to the naked eye. Studies on salinity stress have shown that 3D models can detect very small changes in how leaves lean and roll, which are early indicators for plants to avoid stress. This made it possible to find stress days before chlorosis showed up. This sensitivity highlights the utility of 3D phenotyping as an early warning system in precision farming.

### 3.6. Precision Weed Management and Volumetric Spraying

One of the most significant applications of 3D reconstruction for the environment and the economy is the transition from using broadcast herbicides to targeted, site-specific weed management (SSWM). In “green-on-green” situations, such as weeds growing in crops, traditional 2D vision systems often do not work because the colors are too similar and they block each other. 3D perception addresses these problems by introducing structural and volumetric variations.

**Volumetric Variable-Rate Application (VRA):** 3D reconstruction enables volumetric VRA, which differs from 2D systems that only utilize leaf area index (LAI) to estimate biomass. Using LiDAR or stereo vision to determine the Foliage Area Volume Density (FAVD) or total canopy volume, sprayers can adjust the flow rates of their nozzles using Pulse Width Modulation (PWM) to match the biomass of the target [92]. Studies indicate that volume-based dosage calculation exhibits a significantly stronger correlation (r > 0.83) with weed biomass compared to 2D area measurements. This method prevents the under-dosing of large, erect weeds and decreases chemical waste by 30% to 90%, contingent on weed pressure [93].

**Segmentation by Height and Handling of Occlusion:** In thick canopies, crop leaves or weeds can often block each other. With 3D reconstruction, it is possible to segment based on height, which is an important way to tell the difference between tall weeds (such as Amaranthus palmeri) and shorter crops (such as soybeans) later in the season. LiDAR and SfM-derived Canopy Height Models (CHM) can accurately map these height differences, allowing spot-sprayers to only hit the weed canopy that is above the crop. Also, 3D systems can figure out depth ambiguities that make 2D systems misclassify crop leaves as weeds when they overlap. This reduces crop damage.

**Commercial Use and Latency**: The business world has quickly started using stereo vision to help weeding robots see in 3D. The Carbon Robotics LaserWeeder (Carbon Robotics, Seattle, WA, USA) and other systems utilize high-resolution stereo cameras and strobe lighting to create 3D models of the seedbed in real-time. This volumetric data is necessary for laser ablation to hit the weed’s meristem (growth point) with sub-millimeter accuracy. This is not possible with only 2D centroids [94]. Verdant Robotics (Verdant Robotics, Hayward, CA, USA) employs a “spatial AI” method to create a 4D model (3D + time) of the field, indexing each plant to enable simultaneous weed and fertilization with millimeter accuracy [95]. Latency remains a significant issue, however. Real-time spot spraying at tractor speeds (such as 12 mph) needs 3D data to be processed in less than 300 milliseconds, which is pushing the limits of current edge computing hardware [96].

### 3.7. Strategic Responses to Agriculture-Specific Challenges

Specialized techniques are needed to mitigate environmental dynamics that are not present in controlled laboratory settings when developing reliable 3D reconstruction pipelines for agricultural applications. Creating a robust 3D reconstruction pipeline for the field requires specific techniques to mitigate environmental dynamics, which are often the main reasons models fail.

#### 3.7.1. Wind-Induced Motion Mitigation

The main technical obstacle in open-field settings is wind, which interferes with the static scene assumption that standard feature-matching and triangulation algorithms rely on. To overcome this, recent developments such as 4D Gaussian Splatting (4DGS) add a time-conditioned deformation field to each anisotropic primitive. This enables the system to warp a canonical 3D model to any timestamp, effectively tracking non-rigid motion and swaying leaves [69]. Additionally, Non-Rigid Plant Fusion hybrid pipelines use Non-rigid Iterative Closest Point (NICP) algorithms in conjunction with RGB, depth, and LiDAR data to achieve sub-millimeter accuracy and high temporal stability even when plant structures are moving [97]. Commercial systems also address wind-induced failure through rapid capture rigs and strobe lighting to minimize motion blur, enabling sub-millimeter accuracy for real-time laser ablation at tractor speeds [94].

#### 3.7.2. Strategies for Overcoming Canopy Occlusion

Dense crop canopies’ inherent “self-occlusion” often produces “hollow” 3D models, in which internal stems and fruits are excluded from the final reconstruction. Instead of oversampling easily visible views to increase surface coverage, Self-Supervised Learning Local Next-Best-View (SSL-Local-NBV) algorithms target occluded organs by learning to score candidate viewpoints based on expected information gain. By representing surfaces as continuous functions, neural implicit surface representations such as DeepSDF and Occupancy Networks help to close these gaps and enable the model to precisely fill in plant parts that are obscured or absent from restricted views [60]. Furthermore, by concentrating on a particular Region of Interest, sophisticated neural pipelines such as OB-NeRF facilitate “digital defoliation,” which enables researchers to electronically remove leaf layers to reveal fruit clusters and make highly accurate yield estimates [46]. Lastly, because near-infrared laser pulses can pass through foliage gaps to measure structural traits both above and below the canopy surface, LiDAR is still the gold standard for handling occlusion [29].

### 3.8. Case Studies in Commercial Implementation and Technical Feasibility

High requirements for operational speed and geometric accuracy must be met when moving from theoretical models to commercial agricultural machinery. A standard for evaluating the viability of new 3D reconstruction technologies is provided by examining the technical specifications of top industry solutions.

#### 3.8.1. Autonomous Laser Weeding

The LaserWeeder platform performs real-time sub-millimeter 3D weed localization using an advanced sensor-fusion technique. The system achieves a total data processing latency of less than 100 milliseconds from image acquisition to laser firing, allowing the tractor to run at a steady speed of about 1 to 2 mph. To eliminate motion blur and achieve ±2 mm field accuracy in plant center detection, high-resolution stereo cameras and strobe lighting are employed. High-performance edge computing modules, such as NVIDIA GPUs, perform deep-learning-based segmentation and 3D coordinate mapping in parallel across dozens of crop rows, enabling this quick inference cycle [94].

#### 3.8.2. Precision Spraying and Thinning

Commercial precision spraying and thinning systems, such as the Verdant Robotics system, are built with volumetric accuracy in mind so that chemicals can be applied precisely to the desired target. These systems combine “spatial AI” with a digital twin of the orchard/field, achieving ~1.5 mm precision in modeling each plant’s organs and operational latency of about 30 to 50 ms per frame so that they can accurately trigger nozzles with millisecond precision after receiving a signal to do so. This technical performance enables 3D reconstruction for mapping and for high-speed mechanical and chemical (e.g., applying herbicides) interventions in unconfined (unconstrained) environments [95].

## 4. Challenges and Existing Problems

Even with these improvements, utilizing 3D reconstruction in commercial farming remains challenging. The agricultural environment is “unconstrained,” which goes against the static and controlled assumptions that most computer vision algorithms make. Wind is the biggest technical problem. Standard NeRF, SfM, and MVS all assume that the scene is not moving. Plants, on the other hand, are flexible structures that are always moving. This movement disrupts the geometric consistency required for feature matching. The triangulation algorithm cannot find a convergence point when a leaf moves between frame A and frame B. This causes “ghosting” artifacts, blurring, or the complete deletion of the moving part from the model. This can be fixed with rapid capture rigs, but they are expensive. Wind remains a significant contributor to reconstruction failure in widely used UAV or single-camera ground protocols.

To get the most out of crops, they need to grow densely, but this density makes it hard to see through. When outer leaves cover inner stems and fruit, this phenomenon is known as “self-occlusion.” This creates “hollow” 3D models that lack internal structure. Cameras and other passive sensors cannot put together things they cannot see. This results in biomass and yield being consistently underestimated. Also, thin structures such as peduncles or tendrils are often smaller than the voxel grid or point cloud’s resolution, which can cause parts of the model to be disconnected or “floating.” There is a significant difference between the computing power required by neural rendering and what agricultural robots can achieve. A high-end GPU can take hours to train a NeRF model. While 3D Gaussian Splatting is faster, inference still requires a significant amount of VRAM (often gigabytes), which exceeds the capacity of edge devices such as the NVIDIA Jetson (NVIDIA Corporation, Santa Clara, CA, USA) or Raspberry Pi (Raspberry Pi Foundation, Cambridge, UK) used in field robotics. Sending terabytes of image data to the cloud for processing is often not possible because rural areas do not have good internet access. Right now, this “compute gap” stops high-fidelity 3D reconstruction from being used in real-time field operations and only lets it be used for offline research.

To make AI models, you need data that is labeled. It is, however, very hard to get “ground truth” 3D labels for plants. You cannot easily label every point in a thick cloud as “leaf” or “stem” without spending hundreds of hours doing it by hand. 3D annotation tools are slower and more complicated than 2D bounding boxes. Also, getting geometric ground truth to check the accuracy of the reconstruction is hard because LiDAR has problems with beam divergence and measuring by hand is destructive. This lack of training data makes it challenging for supervised learning methods to function effectively. Lastly, the use of 3D technology is limited by the economy. Farmers have very little room for error and need to see a return on their investment right away. A smartphone-based SfM app is cheap, but it takes a lot of work and is likely to fail. On the other hand, a robotic phenotyping system is dependable, but it is also extremely expensive. There is still a gap in the market for “middle ground” 3D scanning that is strong, cheap, and automatic. Additionally, the data is difficult to interpret, which is a significant issue. Farmers need useful information, such as “spray here,” not just raw point clouds.

## 5. Opportunities and Future Directions

Static 3D reconstruction already addresses many of the issues with 2D phenotyping; however, agriculture is constantly evolving. The next step is 4D Digital Twins, which are spatiotemporal, multimodal, and semantically rich models. They let you predict and act instead of just watching. This section outlines the key directions: 4D Gaussian Splatting, edge-native reconstruction, multimodal fusion, and semantic reconstruction driven by a foundation model.

**From Static Geometry to 4D Spatiotemporal Digital Twins**: Static 3D models, taken at a single point in time, display canopy architecture, but they do not show growth, daily movement, or stress progression. 4D Digital Twins model geometry directly over time, making it possible to perform longitudinal phenotyping and response analysis. 4D Gaussian Splatting (4DGS) is a viable alternative to dynamic NeRFs, as it is accurate but requires a longer training and update time. 3D Gaussian Splatting utilizes explicit Gaussian primitives that can be rendered in real-time. To make this work in 4D, you need to track deformation and create Gaussians to illustrate growth. GrowSplat shows this idea by combining 3D Gaussian Splatting with strong temporal alignment [114]. A two-step registration process that includes coarse feature-based alignment and Fast Global Registration (FGR), followed by fine ICP, keeps the identities of certain structures across time steps. This enables true longitudinal analysis, rather than merely comparing volumes at different points in time. Standard deformation fields are not effective when there are changes in topology, such as when buds emerge or fruit sets. Streaming frameworks, such as Scale-GS, suggest hybrid deformation-spawning strategies that allow existing Gaussians to change shape while new Gaussians are created for structures that become newly visible or are generated. Adding botanical priors to the spawning logic might limit new Gaussians to biologically plausible places, such as axillary meristems and inflorescences. This would reduce artifacts and make predictions more realistic. Table 4 outlines the emerging multimodal fusion strategies and their specific technical approaches for developing agricultural digital twins.

**Lightweight and Edge-Native Architectures**: The current dependence on cloud computing for training neural fields (NeRF) or optimizing Gaussian splats results in a latency bottleneck that is intolerable for real-time robotic intervention. The creation of “Edge-Native” 3D reconstruction algorithms, designed to operate with the limited resources of embedded devices such as the NVIDIA Jetson Orin or Raspberry Pi, presents a significant opportunity [64].

**Model Compression and Distillation**: Techniques such as MobileNeRF and compressed 3D Gaussian Splatting are making memory footprints significantly smaller without compromising the visuals [64]. Future research should concentrate on “distilling” substantial, offline teacher models (e.g., a high-fidelity 3D Gaussian Splatting of a field) into streamlined student models that operate on-board autonomous tractors for obstacle avoidance and crop sensing.

**Co-design of hardware and software**: In agricultural robotics, Neural Processing Units (NPUs) and FPGAs that are optimized for differentiable rendering will probably take the place of general-purpose GPUs. “Smart sprayers” would be able to see 3D volume at 20 mph if they had custom silicon that could rasterize 3D Gaussians at low power. This would make the volumetric VRA discussed in Section 3.6 possible [130].

**Universal Foundation Models**: most of the time, 3D reconstruction pipelines are trained from scratch for certain crops, such as Wheat3DGS and Cotton3DGaussians. This does not work for larger groups. Vision Foundation Models (VFMs), such as Segment Anything (SAM) and DINOv2, are gaining popularity. They could lead to “Universal Plant Models” [26].

**Zero-Shot Generalization**: Future pipelines could produce new crops without requiring retraining by leveraging VFMs’ semantic understanding. For example, a robot that knows how to work with maize should be able to apply its knowledge of structure to sorghum. The MapAnything and VGGT frameworks [18] propose a transition to feed-forward transformers that forecast 3D structure from uncalibrated images in a single iteration, completely eliminating the delicate SfM initialization phase.

**Semantic-Geometric Pre-training**: You can use large sets of synthetic plants (Digital Twins) to pre-train reconstruction networks. A “Large Reconstruction Model” (LRM) for agriculture could learn the basic topology of branching structures in the same way that Large Language Models (LLMs) learn how language works. This would make it strong against the severe occlusions that happen in real fields [80].

**The Invisible Spectrum**: 4D Fusion of Different Modes RGB provides the plant texture, but it does not reveal how the plant’s cells function. The final frontier is combining 3D geometry with imaging techniques that cannot be seen to make a “Transparent Digital Twin.”

**Combining terahertz and X-ray imaging**: New studies on portable X-ray and Terahertz imaging enable visualization of the internal stem density and moisture content without damaging the object. Combining these transmission methods with reflective (RGB/LiDAR) geometry would let breeders look inside the plant and measure traits such as how well it transports water and nutrients in 3D [32].

**Subsurface-Surface Coupling**: Integrating above-ground canopy reconstruction (Photogrammetry) with below-ground root architecture (Ground Penetrating Radar or Rhizotrons) presents a significant challenge. A single model that connects canopy volume to root biomass would alter our understanding of carbon sequestration and drought tolerance [33].

### 5.1. Technical Route Selection Guide for Agricultural Practitioners

The selection of a 3D reconstruction pathway must be guided by a systematic evaluation of the specific agricultural environment, the biological complexity of the target crop, and the required operational throughput. Practitioners should utilize the following expanded framework to balance the fundamental trade-offs between hardware cost, processing latency, and geometric fidelity.

#### 5.1.1. Strategic Recommendations by Operational Scenario

High-Throughput Phenotyping (HTP): For breeding programs requiring sub-millimeter precision for traits such as leaf angle or seed volume, specialized neural models such as OB-NeRF are recommended. These frameworks leverage learned implicit priors to “fill in” occluded regions and achieve an impressive 0.98 mm RMSE relative to ground truth [45].

Real-Time Robotic Intervention: For tasks such as autonomous weeding, thinning, or laser ablation, 3D Gaussian Splatting is the superior choice. It provides the necessary real-time rendering speeds (>150 FPS) and low data processing latency (<100 ms) required for machines to operate at standard tractor speeds [7,42].

Autonomous Navigation and Large-Scale Mapping: LiDAR-Inertial SLAM (e.g., Fast-LIO2 or R3LIVE) remains the gold standard for field mobility. These systems provide absolute scale and maintain trajectory errors below 1 cm, even in feature-poor environments or under varying lighting conditions [83,159].

#### 5.1.2. Adapting to Unconstrained Environmental Challenges

Mitigating Wind and Non-Rigid Motion: In open fields where wind causes constant leaf movement, practitioners should avoid static SfM or standard NeRF pipelines as they result in “ghosting” artifacts. Instead, 4D Gaussian Splatting (4DGS) or Non-Rigid Plant Fusion is recommended to track time-conditioned deformations and swaying structures.

Solving Canopy Occlusion: For dense crops where outer foliage hides internal structures, Active View Planning (SSL-Local-NBV) algorithms are recommended to direct sensors toward high-information viewpoints. Alternatively, LiDAR provides the most reliable penetration of dense canopies, as near-infrared laser pulses can pass through foliage gaps to measure structural traits below the surface.

Managing Varying Illumination: Active sensors such as LiDAR or ToF are preferred for 24/7 operations as they provide their own light source. For RGB-based systems, Plant-NeRF provides specialized shadow-masking and lighting regularization to maintain stability under shifting natural lighting conditions.

#### 5.1.3. Selection by Crop Type and Digital Architecture

Field Crops (such as maize, wheat, and soybeans): For plot-level height and biomass estimation, UAV-based SfM or Smartphone Video SfM provides a cost-effective solution, achieving centimeter-level accuracy (RMSE as low as 2.8 mm) compared to expensive LiDAR benchmarks [150].

Orchards and Fruit Trees: To capture complex branching and fruit clusters with high visual fidelity (PSNR > 34 dB), explicit neural fields (3D Gaussian Splatting) or LiDAR-RGB fusion are recommended. These methods enable “digital defoliation,” allowing researchers to electronically remove leaf layers to estimate yield and fruit volume [110].

Sparse or Legacy Data: In situations where multi-view capture is impossible, Foundation Models (e.g., MapAnything, VGGT) or PixelSplat can infer 3D properties in a single forward pass. This “rapid mapping” capability is ideal for scouting workflows where only a single image per plant is available [82].

## 6. Conclusions

In digital agriculture, the shift from two-dimensional imaging to three-dimensional reconstruction signifies a fundamental paradigm shift from straightforward observation to accurate, volumetric quantification. This review’s synthesis of approaches demonstrates that the emergence of explicit neural representations, such as 3D Gaussian Splatting, has successfully neutralized the long-standing “speed-fidelity trade-off” that previously necessitated a decision between the high cost of LiDAR and the computational latency of earlier neural networks. Modern architectures now provide the first practical route for real-time, on-board volumetric perception by fusing differentiable rendering with the measurable nature of point clouds. This allows robotic systems to extract complex phenotypic traits and carry out targeted interventions without depending on costly active sensors or prohibitive cloud computing. Nevertheless, the analysis shows that no single modality is adequate on its own; “Hybrid Intelligence” architectures exhibit the most reliable performance. Neural approaches offer unmatched visual fidelity and semantic comprehension; however, to ensure metric accuracy in unconstrained field environments, they require the strict constraints of multi-view geometry. Therefore, combining the speed of 3D Gaussian Splatting, the structural accuracy of photogrammetry, and the semantic power of Vision Foundation Models will be the key to agricultural reconstruction in the future. The development of “4D Agricultural Metaverses”, continuously updated, spatiotemporal digital twins that enable autonomous machines to not only perceive crop geometry in real-time but also predict growth and optimize management decisions, is quickly advancing the field and democratizing access to precision agriculture.

## Figures and Tables

**Table 1:** Qualitative Comparison of 3D Reconstruction Methodologies.

Category	Method/Paradigm	Input Modality	Core Principle	Strengths	Limitations	Primary Domains
Geometric Photogrammetry	Structure-from-Motion (SfM) & Multi-View Stereo (MVS)	Multi-view RGB	Feature matching → Bundle adjustment → Dense stereo triangulation	Low-cost, high geometric accuracy (mm-level), flexible workflow	Slow, poor on textureless/reflective surfaces, occlusion issues, scale ambiguity (mono)	Plant Phenotyping, Architecture, Heritage, Low-cost Scanning
Active Sensing & SLAM	LiDAR SLAM & Sensor Fusion	LiDAR, IMU, GPS	Scan matching (ICP) + factor-graph optimization	High geometric accuracy (cm-level), robust lighting invariance, absolute scale, real-time	Expensive hardware, no texture, fails in feature-poor areas	Autonomous Driving, Robotics, Surveying
	Visual SLAM (V-SLAM)	Mono/Stereo/RGB-D, IMU	Real-time tracking via features or photometric error	Low cost, dense texture, real-time	Sensitive to lighting/motion blur; monocular scale drift	AR, Indoor Robotics, UAVs
	Depth-Based Fusion (TSDF/ICP)	RGB-D/Depth sensors	ICP alignment + volumetric TSDF fusion	Real-time dense reconstruction, accurate small-volume geometry	Limited range, sunlight sensitivity, drift without loop closure	Indoor Scanning, Phenotyping Rigs, HCI
	Specialized Active Imaging (Structured Light)	Projected patterns	Phase map reconstruction from structured light	Works in turbid/challenging media, low sampling	Short-range, controlled setups only	Underwater Imaging, Industrial Inspection
Neural Rendering & Reconstruction	Implicit Neural Fields (NeRF)	Multi-view RGB + known poses	Learns continuous radiance field (color + density)	Photorealism, handles occlusion & view-dependence	Slow training/rendering, static scenes, need accurate poses	Novel View Synthesis, VFX, Digital Assets, Complex Canopies
	Explicit Neural Fields (3D Gaussian Splatting)	Multi-view RGB + known poses	Optimizable 3D Gaussians + differentiable rasterization	Real-time (>60 FPS), fast training, editable	Needs SfM poses, floaters, and less surface continuity	VR/AR, Robotics, Digital Twins, Phenotyping
	Hybrid 2D→3D Segmentation	Multi-view RGB	2D semantic segmentation guiding SfM/MVS	High semantic accuracy, cheap annotation, efficient	Errors propagate from 2D → 3D, calibration sensitive	Semantic Phenotyping, Scene Understanding
	Single-View Reconstruction	Single RGB	Learned priors to infer voxel/mesh/implicit shape	One image needed, fast inference, generative	Lower geometric fidelity, prior-dependent, scale ambiguity	Prototyping, AR, Text/Image-to-3D
	Deep MVS	Multi-view RGB + known poses	CNN cost-volume depth regression	More robust than classical MVS, learns priors	High memory, over-smooth detail	General 3D Reconstruction, Benchmarks
	Foundation Models (Transformers)	Multi-view RGB (often uncalibrated)	Pose + geometry prediction in a single forward pass	Fast, robust to sparse/unposed input, strong generalization	Less accurate than optimization-based methods	Rapid Mapping, Pose Estimation, Feature Extraction
	Non-Rigid Fusion	RGB/Depth/LiDAR	Non-rigid ICP + optical flow constraints	Handles deformation/dynamic scenes	High compute, complex calibration	Dynamic Phenotyping, Motion Capture
High-Precision Volumetric	X-Ray CT Reconstruction	CT slice stack	2D slices → 3D volume + marching cubes	Micron-level precision, internal structure	Expensive, lab-only, static specimens	Medical Imaging, Material Science

**Table 2:** Quantitative Performance Benchmarks of 3D Reconstruction Methodologies.

Domain	Method	Representation/Type	Key Performance Metric(s)	Measurement Scenario
Foundation Models	MapAnything	Feed-Forward Transformer	Pose, Depth, Normals, Point Cloud	Rapid field mapping with uncalibrated images (The System does not know camera settings or positions)
	VGGT	Feed-Forward Transformer	Pose, Depth, Normals, Point Cloud	Scalable 3D vision across general large-scale datasets
	DUSt3R/MASt3R	Feed-Forward Transformer	Pointmap/Pose Prediction	Unconstrained capture with mixed focal lengths
Depth-Based Fusion/SLAM	KinectFusion	Volumetric TSDF	Accuracy < 2 mm; Precision < 2 mm; Resolution > 390 pts/cm^2^	Indoor scanning and dense geometric modeling
	LSD-SLAM	Direct Visual SLAM	ATE: 0.03–0.05 m; ~20 FPS	Visual navigation for AR and indoor robotics
	DSO	Direct Visual SLAM	ATE: 0.02–0.04 m; ~30 FPS	Visual navigation for AR and indoor robotics
	ORB-SLAM3	Feature-based Visual SLAM	ATE: 0.015–0.025 m; 25–30 FPS	Autonomous robotics in high-dynamic environments
	LIO-SAM	Hybrid LiDAR-Inertial	ATE: ~0.013–0.04 m; 10–30 FPS	Field navigation on rough terrain mobile rigs
	R3LIVE	Hybrid LiDAR–Vision–Inertial	ATE: 0.009 m; 10 FPS	High-speed SLAM and real-time robotic mapping
	Fast-LIO2/R3LIVE	Hybrid LIO	ATE < 1 cm: >100 FPS (Fast-LIO2)	High-speed SLAM and real-time robotic mapping
	DROID-SLAM	Hybrid Visual SLAM	ATE: 0.010–0.020 m; 15–20 FPS	Visual odometry for benchmark visual navigation
Navigation & SLAM	LORP-Match	Hybrid Feature-Matching	RMSE: 1.7 cm; Precision: 95.4%; 25 FPS	UAV agricultural surveys with low-overlap images
Neural Rendering	Instant-NGP	Transformer + Hybrid 3D Gaussian Splatting	PSNR: 34.2 dB; IoU: 0.81; 180 FPS	Rapid view synthesis on consumer-grade GPUs
	3D Gaussian Splatting (Survey)	Explicit Gaussian Splats	PSNR: 34–36 dB; SSIM: ~0.97; 150–250 FPS; Minutes training	Digital twins and real-time interactive rendering
	NeRF (Surveys)	Implicit MLP	PSNR: 31–35 dB; SSIM: ~0.96; <5 FPS; Hour’s training	Photorealistic assets for high-end VFX/Digital
Neural Reconstruction (Comparative)	HoloRecon (Single-View)	Transformer + Hybrid 3D Gaussian Splatting	PSNR: 34.2 dB; 180 FPS	Single-Shot Reconstruction and novel view generation
	NeRF (Comparative)	Implicit MLP	RMSE: 1.24 mm; IoU: 0.92; Runtime: 180 min	Controlled specimens such as bell pepper reconstruction
	MVSNet	Deep MVS	RMSE: 1.38 mm; IoU: 0.89; Runtime: 36 min	General 3D benchmarks for CNN-based depth
	Metashape	Geometric (Commercial)	RMSE: 1.12 mm; IoU: 0.93; Compl.: 96.7%; 25 min	Standard photogrammetry and low-cost scanning
	SfM + MVS (COLMAP + OpenMVS)	Geometric	RMSE: 1.27 mm; IoU: 0.91; Compl.: 95.1%; 22 min	Standard photogrammetry and low-cost scanning
Plant Phenotyping	OB-NeRF	Implicit MLP	PSNR: 35.7 dB; RMSE: 0.98 mm; R^2^ > 0.97	Complex canopies with hidden plant structures
	Non-Rigid Plant Fusion	Hybrid RGB + Depth + LiDAR + NICP	RMSE: 0.8–1.2 mm; TSI: 0.94	Dynamic plants such as swaying field crops
	2D-to-3D Segmentation	DeepLabv3 + SfM	mIoU: 84.7%	Semantic phenotyping for leaf and stem segmentation
	Smartphone Video SfM	SfM + MVS	R^2^ > 0.99; RMSE: 2.8 mm (vs. LiDAR)	Mobile scouting for smartphone-based traits
	3DPhenoMVS	SfM + MVS	R^2^ > 0.97; Hausdorff < 1 cm (vs. LiDAR)	N/A
	Plant Monitoring Platform	RGB-D + ICP	Registration Error ≤ 1.5 mm	N/A
Sensing (Hardware)	LiDAR (Agri Survey)	TLS/MLS/ALS	TLS: 1–10 mm; MLS: 1–3 cm; ALS: 5–15 cm	Precision agriculture for field and plot monitoring
	Solid-State LiDAR	Active Sensing (OPA/FMCW)	Accuracy: 0.5–3 cm; >200 FPS; <$300	Robotic perception with OPA or FMCW hardware
Materials Science	Asphalt CT-Recon	X-ray CT	RMSE: 0.005–0.009 mm	Micro-structure analysis of asphalt mixtures
Medical Imaging	Reconstruction from JPG	SfM + MVS + Poisson	PSNR: 37.1 dB; SSIM: 0.725	Dental imaging from 2D multi-view radiographs
Underwater Imaging	3D-UFSPI	Fourier Single-Pixel Imaging	PSNR ~17 dB; RMSE ≈ 0.206 px	Turbid water imaging at 50 NTU turbidity
3D Model Retrieval	S2Mix	CNN-based Retrieval	ANMRR: 0.304	N/A

**Table 3:** Decision Matrix for Agricultural 3D Reconstruction Methods.

Methodology	Geometric Accuracy (RMSE/ATE)	Perceptual Fidelity (PSNR)	Speed (Time-to-Model)	Speed (Time-to-View)	Cost	Robustness (Wind)	Robustness (Light/Texture)
SfM/MVS	High (1–3 mm)	Low-Medium	Medium (20–30 min)	Medium	Low	Very Poor (Static Scene)	Very Poor
LiDAR (TLS/MLS)	Very High (1–30 mm)	(Point Cloud)	Fast (Online)	Fast (Points)	Very High	Medium	Excellent
LiDAR-Inertial SLAM	Very High (ATE < 1 cm)	(Point Cloud)	Real-Time (>100 Hz)	Real-Time	Very High	Medium-High	Excellent
NeRF (Specialized)	SOTA (OB-NeRF 0.98 mm)	SOTA (35.7 dB)	Very Slow (Hours)	Very Slow (<5 FPS)	Low	Very Poor (Static Scene)	High
3D Gaussian Splatting	Good (but secondary)	SOTA (34–36 dB)	Fast (Minutes)	SOTA (>150 FPS)	Low	Very Poor (Static Scene)	High
Foundation Models (VGGT)	Good (2–3% of LiDAR)	(Geometry focus)	SOTA (Sub-second)	Real-Time	Low	Poor (Image-based)	Medium-High
Dynamic Fusion (4DGS/NICP)	Very High (0.8–1.2 mm)	High	Slow (Hours)	Real-Time (4DGS)	High	Excellent	Medium

**Table 4:** Emerging Multimodal Fusion Strategies for Digital Twins.

Modality Fusion	Technical Approach	Agricultural Utility
RGB + Hyperspectral	HS-GS: wavelength-encoded spherical harmonics + diffusion priors	Spatial nutrient and disease mapping; “virtual spectrometer”
RGB + Thermal	TGA-GS/thermal texture mapping onto RGB geometry	Water-stress detection, irrigation diagnostics, root-zone health visualization
RGB + LiDAR + Tactile	WildFusion: joint geometry + traversability representation	Navigation in dense canopy, safe manipulation, obstacle avoidance
RGB + Language	Neuro-symbolic SAM/CLIP-style pipelines	Text-queryable 3D scenes, automated scouting, semantic phenotyping

## Data Availability

No new data were created or analyzed in this study. All data and information discussed are available in the referenced literature.
